# Imaging quantum oscillations and millitesla pseudomagnetic fields in graphene

**DOI:** 10.1038/s41586-023-06763-5

**Published:** 2023-11-22

**Authors:** Haibiao Zhou, Nadav Auerbach, Matan Uzan, Yaozhang Zhou, Nasrin Banu, Weifeng Zhi, Martin E. Huber, Kenji Watanabe, Takashi Taniguchi, Yuri Myasoedov, Binghai Yan, Eli Zeldov

**Affiliations:** 1 Department of Condensed Matter Physics, Weizmann Institute of Science, Rehovot, Israel; 2 Departments of Physics and Electrical Engineering, University of Colorado Denver, Denver, CO USA; 3 Research Center for Electronic and Optical Materials, National Institute for Materials Science, Tsukuba, Japan; 4 Research Center for Materials Nanoarchitectonics, National Institute for Materials Science, Tsukuba, Japan

**Keywords:** Electronic properties and materials, Electronic properties and devices

## Abstract

The exceptional control of the electronic energy bands in atomically thin quantum materials has led to the discovery of several emergent phenomena^1^. However, at present there is no versatile method for mapping the local band structure in advanced two-dimensional materials devices in which the active layer is commonly embedded in the insulating layers and metallic gates. Using a scanning superconducting quantum interference device, here we image the de Haas–van Alphen quantum oscillations in a model system, the Bernal-stacked trilayer graphene with dual gates, which shows several highly tunable bands^2–4^. By resolving thermodynamic quantum oscillations spanning more than 100 Landau levels in low magnetic fields, we reconstruct the band structure and its evolution with the displacement field with excellent precision and nanoscale spatial resolution. Moreover, by developing Landau-level interferometry, we show shear-strain-induced pseudomagnetic fields and map their spatial dependence. In contrast to artificially induced large strain, which leads to pseudomagnetic fields of hundreds of tesla^5–7^, we detect naturally occurring pseudomagnetic fields as low as 1 mT corresponding to graphene twisting by 1 millidegree, two orders of magnitude lower than the typical angle disorder in twisted bilayer graphene^8–11^. This ability to resolve the local band structure and strain at the nanoscale level enables the characterization and use of tunable band engineering in practical van der Waals devices.

## Abstract (web summary)

Imaging of quantum oscillations in Bernal-stacked trilayer graphene with dual gates enables high-precision reconstruction of the highly tunable bands and reveals naturally occurring pseudomagnetic fields as low as 1 mT corresponding to graphene twisting by 1 millidegree.

## Main

Determining the band structure (BS) and the Fermi surface is a crucial step in understanding and using the electronic properties of materials. The most sensitive canonical method for mapping the BS of bulk metals and semiconductors is the measurement of the de Haas–van Alphen (dHvA) oscillations^12^. In this quantum mechanical effect, in the presence of magnetic field *B*, electrons coherently circulate in closed electronic orbits, giving rise to quantum oscillations (QOs) in the grand thermodynamic potential *Ω* and in the associated magnetization *M* = −∂*Ω*/∂*B* (ref. ^12^). In two-dimensional (2D) systems, these oscillations are described by the formation of Landau energy levels (LLs) with sharp peaks in the density of states (DOS). Charge carriers orbiting in the metallic LL states give rise to diamagnetic response, whereas ground-state currents flowing in the gapped edge states contribute to paramagnetic magnetization, resulting in magnetization oscillations with either magnetic field or carrier density^12^. As the measured total magnetic moment scales with sample volume, observation of dHvA effect in 2D systems has been challenging^13,14^, in which non-thermodynamic Shubnikov–de Haas (SdH) oscillations are the benchmark characterization tool^15^.

The advances in the fabrication of van der Waals (vdW) atomic layer devices have provided an opportunity for a lot of electronic phases, including tunable correlated insulators^16^, orbital magnetism^17–19^, integer and fractional Chern insulators^20–23^ and unconventional superconductivity^24,25^. Using material selection, stacking order and twist angle, a wide variety of structures with different properties can be engineered. Their BS can be further manipulated through the transverse electric field, magnetic field, strain or pressure. The investigation of the BS in micron-sized vdW devices presently centres on detecting QOs by SdH effect^15,26^ and capacitance^4,25^. However, various types of disorder, such as charge inhomogeneity, twist-angle disorder and strain, are seen in these samples^9,27,28^, and the aforementioned methods lack spatial information. The various inhomogeneities also obscure the QOs in global measurements, requiring the application of elevated magnetic fields to overcome the spatial disorder. Although several scanning probe techniques, including scanning tunnelling microscopy^29,30^ and single-electron transistors^22,31^, are powerful probes of local electronic properties, the former requires the electron layers to be exposed to vacuum as in photoemission studies, and neither of them is suitable for devices encapsulated with a metallic top gate required for applying displacement fields. The development of a tool to measure the local BS in the diverse family of 2D quantum materials is thus highly desirable.

Strain emerges as a particularly intriguing, yet challenging, tunable parameter in vdW devices because of their high mechanical flexibility. In addition to changing the BS and breaking of crystal symmetries, non-uniform strain creates pseudomagnetic fields (PMFs) because of the valley degree of freedom in hexagonal vdW materials^32^. PMFs of tens to hundreds of teslas have been observed in artificially strained graphene nanostructures^6,7^. These gauge fields create effective LLs with sharp peaks in DOS that strongly vary in space and alter the electronic transport properties^5^. Yet, PMFs stemming from the natural strain formed during the fabrication process have remained unknown.

**Fig. 1:**
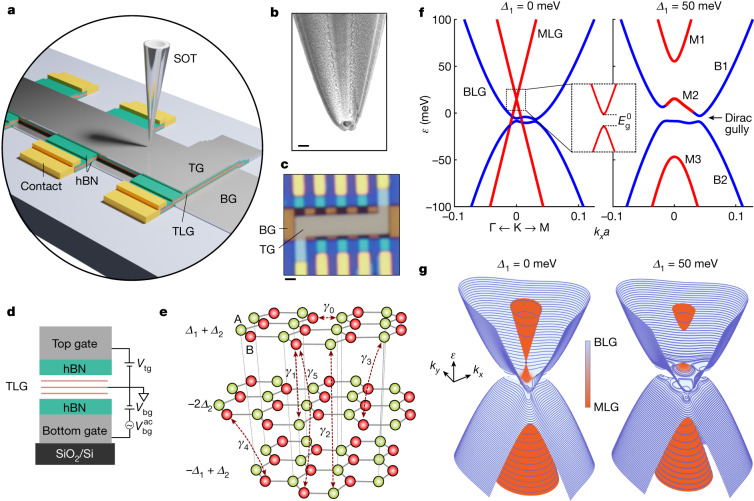
Experimental setup and ABA graphene BS. **a**, Schematic sample structure with TLG encapsulated by hBN and top (TG) and bottom (BG) Pt gates, with scanning SOT. **b**, Scanning electron microscope image of the indium SOT. **c**, Optical image of the TLG device. **d**, The stacking geometry of the device with indicated *V*_tg_ and *V*_bg_ voltages applied to the top and bottom gates, respectively, for controlling the carrier density *n* and displacement field *D*. **e**, Atomic structure of ABA graphene with indicated SWMc parameters. **f**, The BS of ABA TLG for zero displacement field (*Δ*_1_ = 0 meV) and for *Δ*_1_ = 50 meV. Inset, a small Dirac gap ${E}_{{\rm{g}}}^{0}$ is present in the MLG band at *Δ*_1_ = 0 meV, which grows rapidly with *Δ*_1_. **g**, Three-dimensional rendering of the BS reconstructed from the dHvA oscillations with overlaid contours of the calculated LLs. The LLs are shown for *B*_a_ = 1 T for clarity. At our *B*_a_ = 320 mT, the LLs are three times denser. The colour map represents the wavefunction projection onto the MLG-like (red) and BLG-like (blue) bands. Scale bars, 200 nm (**b**) and 1 μm (**c**).

Using scanning SQUID (superconducting quantum interference device)-on-tip (SOT) microscopy^33^, we imaged the dHvA effect in hBN-encapsulated dual-gated Bernal-stacked trilayer graphene (TLG) (Fig. 1a). The high magnetic sensitivity of the SOT enables imaging of the QOs at low fields resolving the multi-band electronic structure with sub-meV energy resolution and unperturbed by elevated magnetic fields. The quantitative information provided by the thermodynamic oscillations enables high-precision derivation of the tight-binding hopping parameters and accurate reconstruction of the tunable band hybridization induced by the displacement field. Moreover, the nanoscale spatial resolution enables a detailed quantitative study of the spatial variations of the QOs over the entire device, showing the presence of PMFs of millitesla magnitude in micron-sized domains.

## BS of ABA graphene

The Bernal-stacked ABA TLG is the minimal graphene structure requiring the full set of parameters in the Slonczewski–Weiss–McClure (SWMc) tight-binding model^34^ (Fig. 1e) with six hopping parameters *γ*_*i*_ (*i* = 0–5), on-site energy difference *δ* due to stacking, potential difference *∆*_1_ between the adjacent graphene layers induced by the applied displacement field *D* and potential difference *∆*_2_ that describes the non-uniform charge distribution between the middle and the outer layers. The influence of these parameters on the BS is shown in Extended Data Fig. 4.

Owing to the mirror symmetry of the crystal, the bands decompose into a monolayer-graphene (MLG)-like band with Dirac dispersion and a bilayer-graphene (BLG)-like quadratic band, with an energy shift between them (Fig. 1f, left). The *D* field breaks this symmetry, leading to band hybridization and Lifshitz transitions with multiple changes in the band topology. At high *D*, the Dirac band divides into three sections (Fig. 1f, right), with M1 and M3 sections separated from the BLG bands B1 and B2, whereas M2 merges with B1 and evolves into gapped mini-Dirac cones at the bottom of B1 (Fig. 1g). These cones (gullies) exhibit three-fold rotational symmetry leading to various possible quantum Hall ferromagnetic and nematic states^35,36^.

Previous studies have explored SdH and capacitance oscillations in TLG at elevated fields^3,37,38^ to determine the BS and identify broken-symmetry states^4,39,40^, yielding a wide span of derived SWMc parameters (Extended Data Table 1), with details of the BS still under debate. In particular, the size and polarity of the Dirac gap, ${E}_{{\rm{g}}}^{0}$, at *D* = 0 (Fig. 1f, inset) is controversial^40^. Moreover, it was predicted that the trigonal warping induced by *γ*_3_ breaks the rotational symmetry with notable consequences on the LL structure, resulting in LL anticrossings, which occur between a given MLG LL and every third BLG LL^36,41^. Although some single anticrossings were reported^40^, the predicted periodicity has not been observed directly. Moreover, symmetry breaking leading to gully-polarized states has been reported in high magnetic fields^4^, but gully coherence at low fields remains an open question.

## Nanoscale magnetic imaging and results

**Extended Data Fig. 1:**
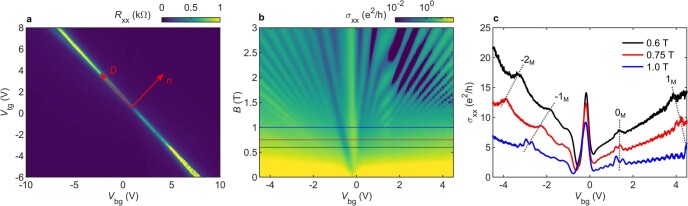
Transport characterization of ABA graphene. **a**, A dual-gate sweep measurement of ${R}_{{xx}}$ at $T=$ 200 mK in ${B}_{a}=$ 0 T in device A described in the main text. **b**, The Landau fan of ${\sigma }_{{xx}}$ in device B at $T=$ 1.67 K. **c**, The Shubnikov–de Haas oscillations in ${\sigma }_{{xx}}$ at 0.6, 0.75 and 1.0 T along the lines marked in **b** with indicated MLG LLs. The BLG LLs are visible only at ${B}_{a}\,\gtrsim$ 0.75 T for electron doping.

The TLG was encapsulated by approximately 30 nm hBN with top and bottom Pt gates for controlling the carrier density *n* and displacement field *D* (Fig. 1 and Methods). Transport measurements of *R*_*xx*_ versus *n* and applied magnetic field *B*_a_ show a Landau fan with several LL crossings (Extended Data Fig. 1), similar to those in previous reports^40^. Local dHvA oscillations measurements were performed using indium SOT of 150 nm diameter at a height of *h* ≈ 150 nm above the graphene at *T* ≈ 160 mK (Fig. 1b and Methods). A small a.c. voltage ${V}_{{\rm{bg}}}^{\mathrm{ac}}$ at about 1.8 kHz modulates *n* by *n*^ac^, inducing a local a.c. magnetic field ${B}_{{\rm{z}}}^{\mathrm{ac}}$ recorded by the scanning SOT. This ${B}_{{\rm{z}}}^{\mathrm{ac}}$ reflects the differential change *m*_*z*_ in the local orbital magnetization *M*_*z*_, *m*_*z*_ = ∂*M*_*z*_/∂*n*.

**Fig. 2:**
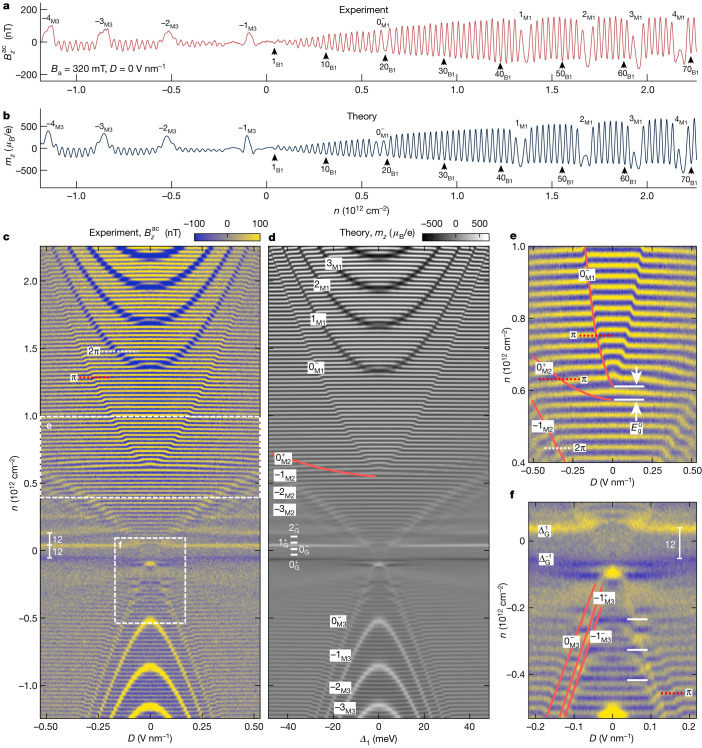
Measurement of dHvA effect in ABA graphene. **a**, The measured local magnetic QOs signal ${B}_{{\rm{z}}}^{\mathrm{ac}}$ as a function of *n* at 160 mK at a fixed location in the interior of the sample at *B*_a_ = 320 mT and *D* = 0 V nm^−1^ using ${V}_{{\rm{b}}{\rm{g}}}^{{\rm{a}}{\rm{c}}}=8\,{\rm{m}}{\rm{V}}$ rms. Some indices of the LLs in M1, B1 and M3 bands are indicated. **b**, The calculated dHvA differential magnetization *m*_*z*_ at *D* = 0 V nm^−1^ using the derived BS. **c**, The measured ${B}_{z}^{{\rm{a}}{\rm{c}}}$ versus *n* and *D*. Crossings between the four-fold degenerate BLG LLs (horizontal yellow and blue lines) and the zeroth K^−^ valley MLG LL, ${0}_{{\rm{M1}}}^{-}$, results in a π shift (red dotted line), whereas crossing with the higher MLG LLs introduces a 2π shift (white dotted line). The white vertical bars indicate the 12-fold degeneracy of the LLs in the gullies. **d**, Calculated *m*_*z*_(*n*, *D*) using the fitted SWMc tight-binding parameters with Dingle broadening of 0.3 meV. The MLG LLs in M1, M2 and M3 bands and the gully LLs are labelled. **e**, Magnification of the measured ${B}_{z}^{{\rm{a}}{\rm{c}}}\,(n,D)$ in the vicinity of MLG Dirac gap *E*_g_ (dashed rectangle in **c**) with marked MLG LLs (red). The π shifts at crossings between BLG and ${0}_{{\rm{M2}}}^{+}$ and ${0}_{{\rm{M1}}}^{-}$ valley-polarized MLG LLs and 2π shift at crossing of valley degenerate ${-1}_{{\rm{M2}}}$ LL are indicated. **f**, Magnification of ${B}_{z}^{{\rm{a}}{\rm{c}}}\,(n,D)$ near the top of the BLG valence band B2 showing anticrossings between the MLG and BLG LLs. The white horizontal bars indicate enlarged anticrossing gaps for every third BLG LL (Extended Data Fig. 6).

**Extended Data Fig. 2:**
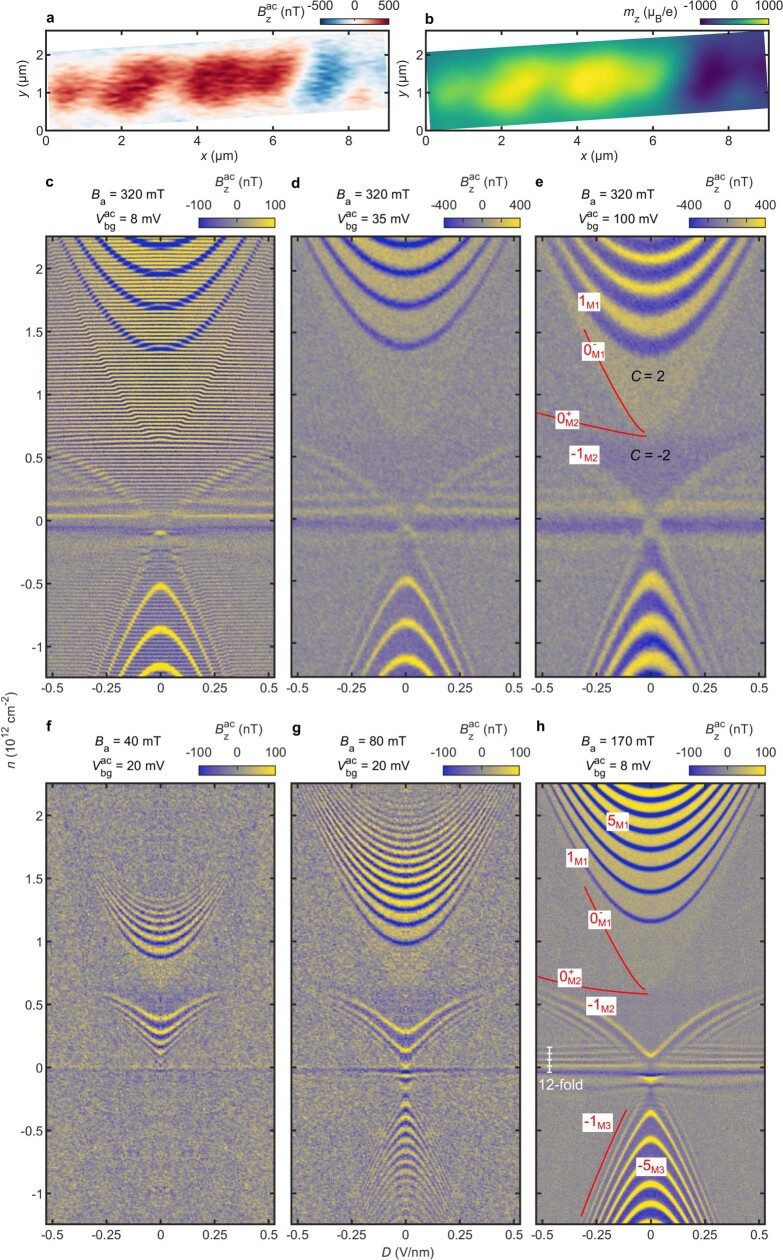
Reconstruction of the local magnetization from ${{\boldsymbol{B}}}_{{\boldsymbol{z}}}^{{\boldsymbol{a}}{\boldsymbol{c}}}({\boldsymbol{x}},{\boldsymbol{y}})$ and comparison of ${{\boldsymbol{B}}}_{{\boldsymbol{z}}}^{{\boldsymbol{a}}{\boldsymbol{c}}}({\boldsymbol{n}},{\boldsymbol{D}})$ at different magnetic fields and ${{\boldsymbol{V}}}_{{\boldsymbol{b}}{\boldsymbol{g}}}^{{\boldsymbol{a}}{\boldsymbol{c}}}$. **a**, Example of the measured ${B}_{z}^{{ac}}\left(x,y\right)$ at *n* = 5.16 × 10^12^ cm^−2^, ${B}_{a}$ = 320 mT, and ${V}_{{bg}}^{{ac}}$ = 10 mV rms. **b**, Differential magnetization ${m}_{z}=\partial M/\partial n$ reconstructed from **a**. **c**, The measured QOs at ${B}_{a}$ = 320 mT and ${V}_{{bg}}^{{ac}}$ = 8 mV rms. The induced peak-to-peak carrier density modulation, ${n}^{{ac}}$ = 1.47 × 10^10^ cm^−2^, is about half of the LL degeneracy $4{B}_{a}/{\phi }_{0}$ allowing clear resolution of the BLG and MLG LLs. **d**, QOs at ${B}_{a}$ = 320 mT and ${V}_{{bg}}^{{ac}}$ = 35 mV rms. The BLG LLs are washed out by the large carrier modulation while MLG LLs and the 12-fold degenerate LLs in the gullies are well resolved. **e**, At ${V}_{{bg}}^{{ac}}$ = 100 mV rms the MLG QOs are smeared out. The paramagnetic response in the MLG LL gaps with Chern numbers $C=\pm 2$ are clearly visible as indicated. **f**, QOs at ${B}_{a}$ = 40 mT and ${V}_{{bg}}^{{ac}}$ = 20 mV rms. At this low field most of the LLs are smeared by the intrinsic LL broadening and only the lowest MLG LLs in sections M1 and M2 can be resolved at low displacement fields. **g**, Same as **f** at ${B}_{a}$ = 80 mT. **h**, At ${B}_{a}$ = 170 mT and ${V}_{{bg}}^{{ac}}$ = 8 mV rms the BLG LLs cannot be resolved, but all the MLG LLs and the 12-fold degenerate LLs in the gullies are very prominent.

Figure 2c shows dHvA oscillations acquired at *B*_a_ = 320 mT at a single point above the sample versus *D* and low carrier densities *n* between −1.2 × 10^12^ cm^−2^ and 2.3 × 10^12^ cm^−2^. A line cut of the data at *D* = 0 V nm^−1^ is shown in Fig. 2a. Notably, in this relatively small *n* range, we observe more than 100 LLs, in sharp contrast to transport measurements (Extended Data Fig. 1), in which no SdH oscillations can be discerned at such low *B*_a_. Moreover, we can resolve dHvA oscillations at fields as low as 40 mT (Extended Data Fig. 2). To our knowledge, this is the lowest *B*_a_ at which QOs have been observed in 2D systems. As shown in Fig. 1g, mapping these dense LLs offers a distinctive quantitative approach for high-precision BS reconstruction and derivation of high-accuracy SWMc parameters as summarized in Extended Data Table 1.

Figure 2b,d shows dHvA oscillations calculated from the fitted BS, showing remarkable qualitative and quantitative agreement with the experiment. Such accurate reconstruction is possible because the thermodynamic QOs can be calculated quantitatively from the BS (Methods). The observed QOs can be classified by five sets of LLs.

In the B1 and B2 bands, as the DOS in the BLG bands is much higher than in MLG bands, the LLs in B1 and B2 bands appear as dense horizontal lines in Fig. 2c,d. At low fields, the LLs are four-fold valley and spin degenerate, dispersing as approximately $\pm \sqrt{{n}_{{\rm{B}}}\left({n}_{{\rm{B}}}-1\right)}{B}_{{\rm{a}}}$ where *n*_B_ is the BLG LL index. At *B*_a_ = 320 mT, the energy spacing between the B1 LLs is about 1 meV and 0.6 meV in B2, defining our energy resolution of better than 0.6 meV.

In the M1 band, the MLG LLs disperse as approximately $\pm \sqrt{\left|{n}_{{\rm{M}}}\right|{B}_{{\rm{a}}}}$ and are much sparser because of the low DOS. Displacement field opens a large gap *E*_g_ between the MLG sections (Fig. 1f,g) resulting in parabolic-like upturn of M1 LLs with *D* in Fig. 2c,d. At LL crossings, B1 LLs show a phase shift because an M1 LL has to be filled before subsequent B1 LLs can be occupied, with the shift magnitude determined by the LL degeneracy. The high M1 LLs are four-fold degenerate, causing a 2π phase shift as indicated by the dotted white line in Fig. 2c,e. Owing to the topological nature of the Dirac point, the zeroth MLG LLs are valley polarized with ${0}_{{\rm{M1}}}^{-}$ LL (zeroth LL in K^*−*^ valley) residing at the bottom of the M1 band, whereas ${0}_{{\rm{M2}}}^{+}$ LL (zeroth LL in K^+^ valley) is pinned to the top of the M2 band. As these two zeroth LLs are two-fold spin degenerate^36,41^, their crossing with the four-fold degenerate B1 LLs results in a π rather than a 2π shift (Fig. 2c,e, red dotted lines). Moreover, higher M1 LLs show a pronounced negative (dark) diamagnetic signal^42^. By contrast, the ${0}_{{\rm{M}}1}^{-}$ and ${0}_{{\rm{M}}2}^{+}$ LLs in Fig. 2c–e are invisible with their presence discerned by only B1 LLs phase shift. This arises from the Berry phase pinning of the zeroth LL compressible states to band extrema with zero kinetic energy and hence no diamagnetism. However, the incompressible states in the MLG LL gaps show a paramagnetic response^42^ determined by the Chern number *C* as shown in Extended Data Fig. 2e.

In the M2 band, the band hybridization results in a small M2 hole pocket with the total DOS that is too low to accommodate even a single LL at elevated *B*_a_. Consequently, the M2 LLs could not be identified previously^4,37,40^. Our low *B*_a_ and high sensitivity enable clear resolution of M2 LLs (Fig. 2c–e and Extended Data Fig. 2). Figure 2e also shows a gap ${E}_{{\rm{g}}}^{0}$ between the ${0}_{{\rm{M1}}}^{-}$ and ${0}_{{\rm{M2}}}^{+}$ LLs at *D* = 0, comparable to the gap between the two B1 LLs (about 1 meV). The zeroth LLs rapidly separate with *D*, indicating that *E*_g_ grows continuously without intermediate gap closure, contrary to previous suggestions^4,40^.

In the M3 band, at elevated hole doping, the M3 LLs mirror the behaviour of M1 LLs. At low doping, in contrast, the strong hybridization between M3 and B2 bands induces unusual valley polarization. This is demonstrated in Fig. 2f, in which the four-fold degenerate −1_M3_ LL splits into valley polarized ${-1}_{{\rm{M3}}}^{+}$ and ${-1}_{{\rm{M3}}}^{-}$ LLs, accompanied by multiple LL crossings and anticrossings (Extended Data Fig. 6). In particular, avoided crossings with every third BLG LL have been predicted^36,41^ because of trigonal warping. This triple period, unidentified so far, to our knowledge, is resolved in our data (white bars in Fig. 2f) in agreement with the calculations in Extended Data Fig. 6. We also resolve the ${0}_{{\rm{M3}}}^{-}$ LL with no diamagnetism, which induces a π shift in the B2 LLs (Fig. 2f, red dotted line).

**Extended Data Fig. 3:**
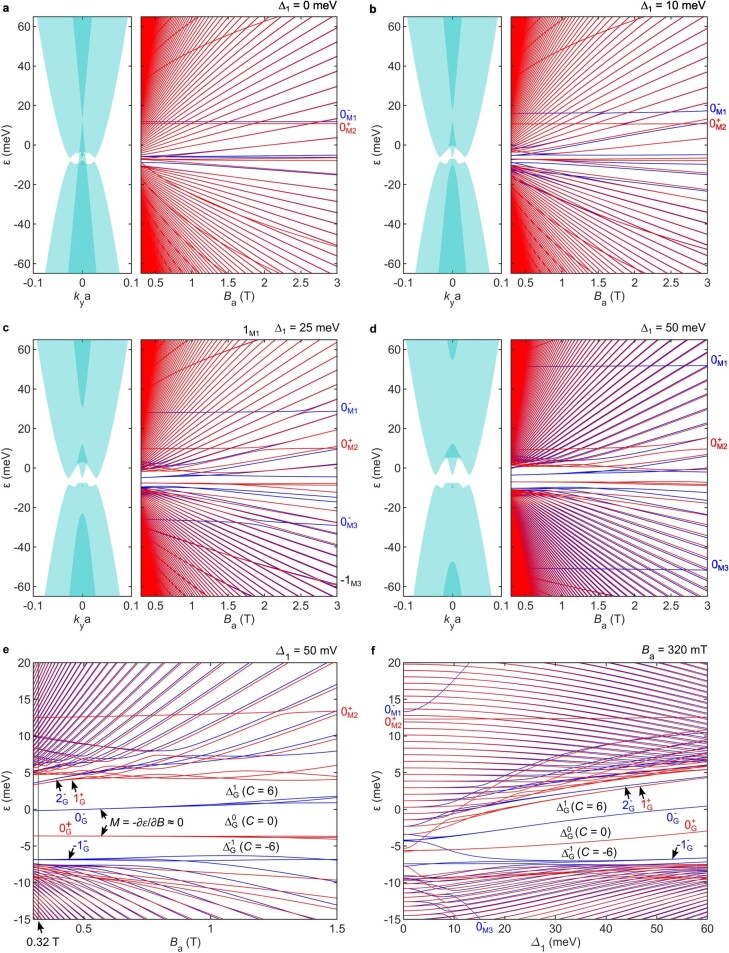
Evolution of ABA graphene band structure with displacement field. **a**–**d**, Projected 3D band structure of ABA graphene (left panels) and the corresponding evolution of LLs with ${B}_{a}$ at ${\triangle }_{1}$ = 0, 10, 25, and 50 meV (right panels). Red and blue lines denote the LLs in ${{\rm{K}}}^{+}$ and ${{\rm{K}}}^{-}$ valleys respectively. The tight-binding parameters used for the calculations are given in Extended Data Table 1 (bottom row). With increasing ${\triangle }_{1}$, the MLG and BLG bandgaps grow and band hybridization is enhanced forming mini-Dirac cones (gullies) near CNP. **e**, Zoomed-in view of the evolution of LLs with ${B}_{a}$ at ${\triangle }_{1}$ = 50 meV. At low fields the zeroth LLs ${0}_{G}^{+}$ and ${0}_{G}^{-}$ are six-fold degenerate (including spin) and have vanishing magnetization. The six-fold degeneracy of the gully LLs is partially lifted at high ${B}_{a}$. The Chern numbers $C$ in the large gaps are indicated. **f**, Evolution of LLs with ${\triangle }_{1}$ at ${B}_{a}$ = 320 mT.

In LLs in the gullies, near charge neutrality point (CNP), on increasing *D*, the enhanced band hybridization and trigonal warping results in three-fold rotationally symmetric Dirac gullies^36,41^ with highly intriguing LL evolution. The low-energy BLG LLs, which are mostly valley degenerate at *D* = 0, undergo valley polarization and intertwining, forming valley-polarized six-fold degenerate LLs in the gully pockets (Extended Data Fig. 3f). The zeroth gully LLs, ${0}_{{\rm{G}}}^{-}$ and ${0}_{{\rm{G}}}^{+}$, exhibit no diamagnetism and the ${\Delta }_{{\rm{G}}}^{0}$ gap between them has *C* = 0. Consequently, a magnetism-free strip of width corresponding to 12-fold degeneracy (${0}_{{\rm{G}}}^{-}$, ${0}_{{\rm{G}}}^{+}$ and ${\Delta }_{{\rm{G}}}^{0}$) is observed around the CNP in Fig. 2c,d,f at elevated *D*. The positive and negative (yellow and blue) signals outside the strip are the paramagnetic responses in the LL gaps ${\Delta }_{G}^{1}$ and ${\Delta }_{{\rm{G}}}^{-1}$ (Fig. 2f and Extended Data Fig. 3e,f).

The marked consistency between the experimental data (Fig. 2c) and the single-particle BS calculations (Fig. 2d) across the entire (*n, D*) plane suggests that the electron–electron interactions play a negligible part in ABA graphene at low *B*_a_ and that the band parameters do not vary in our accessible parameter range. Our dHvA imaging technique is also applicable to moiré systems as demonstrated in Extended Data Fig. 9 for twisted double bilayer graphene, showcasing intricate crossings between LLs in flat and dispersive bands, which can provide indispensable information for the study of correlation effects.

## LL interferometry and strain-induced PMF

**Fig. 3:**
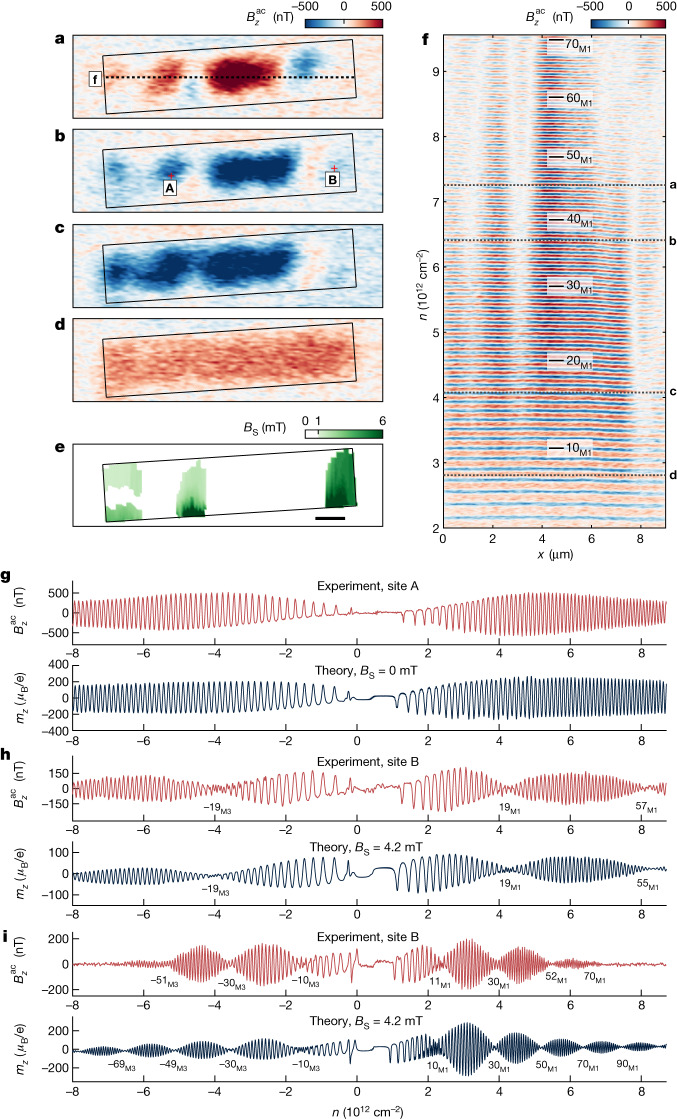
Imaging the beating of QOs and mapping the PMFs. **a**–**d**, Spatial imaging of ${B}_{z}^{{\rm{a}}{\rm{c}}}(x,y)$ at *B*_a_ = 320 mT and *n* = 7.26 × 10^12^ cm^−2^ (**a**), 6.41 × 10^12^ cm^−2^ (**b**), 4.08 × 10^12^ cm^−2^ (**c**) and 2.81 × 10^12^ cm^−2^ (**d**) corresponding to dotted lines in **f**. The black rectangle indicates the boundaries of the TLG. **e**, Map of the derived PMF *B*_S_ across the sample. Regions with *B*_S_ below our resolution of 1 mT are shaded in white. **f**, Line scans of ${B}_{z}^{{\rm{a}}{\rm{c}}}(x)$ versus *n* measured along the dotted line marked in **a** showing QOs from the MLG LLs in the M1 band. **g**, The measured QOs due to MLG LLs (top) at location A indicated in **b** and the calculated QOs (bottom). **h**, The measured QOs (top) at location B and the calculated QOs (bottom) with *B*_S_ = 4.2 mT. The LL indices at the beating nodes are indicated. **i**, Same as **h** at *B*_a_ = 170 mT. The applied larger ${V}_{{\rm{b}}{\rm{g}}}^{{\rm{a}}{\rm{c}}}\,=10\,{\rm{m}}{\rm{V}}$ rms in **a**–**d** and **i**, and 20 mV rms in **f**–**h** averages out the QOs due to BLG LLs intensifying the visibility of MLG LLs. Scale bar, 1 μm (**e**).

Next, we analyse QOs over the full range of accessible carrier densities |*n*| ≲ 9 × 10^12^ cm^−2^, which enables resolving much finer details of the BS and its spatial dependence. Because at *B*_a_ = 320 mT in this *n* range there are about 500 BLG and more than 100 MLG LLs, we focus on the sparser MLG LLs by applying a larger ${V}_{{\rm{b}}{\rm{g}}}^{{\rm{a}}{\rm{c}}}$ (Methods). Figure 3a–d shows the spatial dependence of ${B}_{z}^{{\rm{a}}{\rm{c}}}(x,y)$ at several densities, whereas Fig. 3f presents ${B}_{z}^{{\rm{a}}{\rm{c}}}(x)$ versus *n* along the dotted line in Fig. 3a. For |*n*| ≲ 3 × 10^12^ cm^−2^, shown in Fig. 2, the QOs exhibit relatively high spatial uniformity as shown in Fig. 3d and at the bottom of Fig. 3f, demonstrating high sample quality. At higher *n*, however, distinctly different behaviour is observed depending on the location as demonstrated in Fig. 3g,h showing the QOs at sites A and B indicated in Fig. 3b. Large parts of the sample, exemplified by site A, show continuous evolution of QOs (Fig. 3g), consistent with the calculations. In other parts of the sample as site B, however, striking low-frequency beating of the MLG LLs is found (Fig. 3h). At a lower *B*_a_ = 170 mT, the beating nodes are shifted to lower LL indices (Fig. 3i).

As the MLG and BLG bands have very different dispersions, the beating cannot arise from their interference. It must therefore originate from small symmetry breaking between the four flavours of the MLG band. In the Methods, we consider various possible mechanisms, including staggered substrate potential, Kekulé distortions, band shifting, Zeeman effects and spin–orbit coupling, as well as non-symmetry-breaking disorder, and show that they are inconsistent with the observed behaviour. Below, we demonstrate that the interference of the QOs is well described by strain-induced PMF (*B*_S_).

**Fig. 4:**
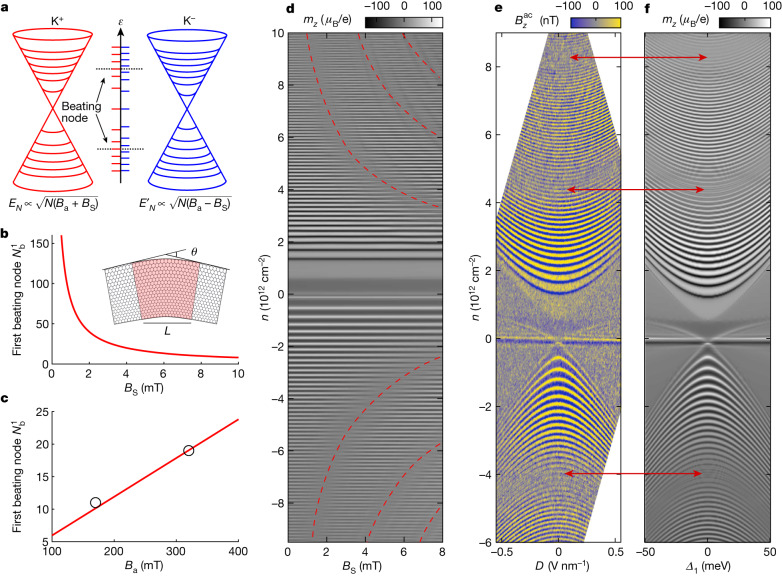
LL interferometry of strain-induced PMF. **a**, Schematic of LL beating in the presence of PMF (*B*_S_) in graphene. Only the MLG Dirac bands with LLs are shown for clarity. **b**, The LL index of the first beating node ${N}_{{\rm{b}}}^{1}$ in the Dirac band versus *B*_S_ at *B*_a_ = 320 mT. Inset, schematic of a graphene strip with arc-like bent section (red) of length *L* and bend angle *θ* generating strain-induced *B*_S_. The illustrated *θ* is greatly exaggerated compared with the maximal derived *θ* ≈ 6 × 10^−3^ degrees. **c**, Calculated dependence of ${N}_{{\rm{b}}}^{1}$ on *B*_a_ for *B*_S_ = 4.2 mT (red). The open circles show the measured ${N}_{{\rm{b}}}^{1}$ at *B*_a_ = 170 mT and 320 mT. **d**, Calculated QOs in the MLG band versus *n* and *B*_S_ at *B*_a_ = 320 mT and *D* = 0 V nm^−1^. The locations of the beating nodes ${N}_{{\rm{b}}}^{1}$ to ${N}_{{\rm{b}}}^{3}$ are highlighted in red. **e**, The measured QOs in the MLG bands versus *n* and *D* using ${V}_{{\rm{b}}{\rm{g}}}^{{\rm{a}}{\rm{c}}}=20\,{\rm{m}}{\rm{V}}$ rms showing beating nodes (red arrows). **f**, Calculated QOs versus *n* and *D* at *B*_a_ = 320 mT and *B*_S_ = 4.2 mT.

Long-wavelength mechanical strain induces an effective gauge field in graphene, with opposite signs for the two valleys. Isotropic or uniaxial strains yield zero PMF, whereas non-uniform shear strain produces a finite *B*_S_ (ref. ^32^). In the presence of *B*_a_, carriers in the K^+^ and K^−^ valleys experience effective fields *B*_eff_ of *B*_a_ + *B*_*S*_ and *B*_a_ *−* *B*_*S*_, inducing a relative shift in the LLs and interference (Fig. 4a). In the MLG Dirac band, the LL energies are given by $${E}_{N}=\sqrt{2e\hbar {v}_{{\rm{F}}}^{2}{B}_{{\rm{eff}}}N}$$, where *e* is the elementary charge, *ħ* is the reduced Plank constant and *v*_F_ is the Fermi velocity. Hence for *B*_S_ ≪ *B*_a_, the energy shift between the LLs in the two valleys is $$\delta {E}_{N}=\sqrt{2e\hbar {v}_{{\rm{F}}}^{2}N}\left(\sqrt{{B}_{{\rm{a}}}+{B}_{{\rm{S}}}}-\sqrt{{B}_{{\rm{a}}}-{B}_{{\rm{S}}}}\right)\approx {B}_{{\rm{S}}}\sqrt{2e\hbar {v}_{{\rm{F}}}^{2}N/{B}_{{\rm{a}}}}$$. Because *δE*_*N*_ scales with √*N*, the lowest LLs remain almost degenerate. For higher LLs, the relative shift between the valley-polarized LLs grows continuously with energy, resulting in beating. The first destructive interference occurs when *δE*_*N*_ = Δ*E*_*N*_/2, where $$\Delta {E}_{N}={E}_{N+1}-{E}_{N}\,\approx $$$$\sqrt{e\hbar {v}_{{\rm{F}}}^{2}{B}_{{\rm{a}}}/2N}$$ is the LL energy spacing, resulting in the first beating node at $$N={N}_{{\rm{b}}}^{1}={B}_{{\rm{a}}}/4{B}_{{\rm{S}}}$$.

Figure 4b shows the theoretical $${N}_{{\rm{b}}}^{1}$$ dependence on *B*_S_, and the calculated beating patterns of the QOs versus *B*_S_ are presented in Fig. 4d. At the nodes, the K^+^ and K^−^ LLs are out of phase, resulting in amplitude suppression and a barely visible frequency doubling. In Fig. 3h (red curve), we observe the first node at $${N}_{{\rm{b}}}^{1}=19$$, yielding $${B}_{{\rm{S}}}={B}_{{\rm{a}}}/4{N}_{{\rm{b}}}^{1}\,=$$$$4.2\,{\rm{mT}}$$. The calculated QOs with *B*_S_ = 4.2 mT (black curve) show an excellent agreement with the data and also well reproduce the secondary nodes at −19 and 57. Moreover, the observed evolution of the interference with *D* (Fig. 4e) is well reproduced by the simulations (Fig. 4f), both in the position of all the three beating nodes (red arrows) and in their evolution with *D* using a single fitting parameter *B*_S_. Because the BS changes profoundly with *D* and the strain-induced PMF should be independent of the BS, the fact that the observed beating is well described by a *D*-independent *B*_S_ provides extra strong support for the model. Furthermore, the PMF should affect all the bands. An important self-consistency check is, therefore, the observation of beating also in the BLG LLs.

Extended Data Fig. 7 shows a closer examination of the BLG QOs at the same location B. Beating is observed and well reproduced by simulations using the same set of parameters. Finally, a crucial test of the PMF origin of the interference is the predicted linear dependence of ${N}_{{\rm{b}}}^{1}$ on *B*_a_, distinguishing it from other possible mechanisms (Methods). Figure 3i (red curve) presents the LL interference measured at *B*_a_ = 170 mT, showing several beating nodes. The calculated QOs (black curve) show an excellent agreement with the data, confirming the linear dependence (Fig. 4c).

Analysing the LL interference over the entire sample, we derive a map of *B*_S_ (Fig. 3e). In large regions, *B*_S_ is below our resolution of about 1 mT, set by the highest accessible *n* (Methods). We find four regions with characteristic length *L* ≈ 1 µm with smoothly varying *B*_S_ reaching up to 6 mT. Several types of lattice distortion—finely tuned triaxial strain, arc-like in-plane bending or stretching a trapezoid-like geometry—have been shown theoretically to produce relatively homogenous *B*_S_ (refs. ^5,43,44^). In our sample, the strain probably arises because of the in-plane bending introduced during the fabrication processes (Fig. 4b, inset). An arc segment of length *L* with a twist angle *θ* in a graphene strip generates ${B}_{{\rm{S}}}\approx c\beta \frac{{\phi }_{0}}{aL}\theta$ (ref. ^43^), where *a* = 0.14 nm is the graphene interatomic distance, *c* ≈ 1 is a numerical constant, *β* ≈ 2 describes the hopping parameter dependence on *a*, *ϕ*_0_ = *h*/*e* is the flux quantum and *h* is the Planck constant. The measured 1 < *B*_S_ < 6 mT thus corresponds to twisting by 1 < *θ* < 6 millidegrees, or equivalently bending radius of 1 < *R* < 6 cm and corresponding strain of 8 × 10^−6^ < *ū* < 5 × 10^−5^, where *R* = *L*/*θ* and *ū* = *θ*/2 (ref. ^43^). These minute bending angles and strains should be abundant in exfoliated atomic layer devices. Strains of larger orders of magnitude and angle disorder have been reported to occur naturally in twisted and stacked graphene structures^8–11,30,45^.

## Discussion

The local dHvA QO technique developed provides a tool for high-precision, quantitative reconstruction of the BS in 2D materials and of its spatial dependence at the nanoscale level. Unlike global measurements, which are affected by spatial inhomogeneities and strain, the local dHvA effect offers high energy resolution approaching the limit of intrinsic lifetime broadening of the energy bands. Moreover, the magnetic QOs are not obscured by metallic gates and multilayer structures, enabling investigation of most of the state-of-the-art vdW devices with in situ tunable BS. Most importantly, the method is not limited to single-particle physics and can show the BS governed by many-body effects and strong interactions in flat-band materials. It can thus improve our modelling and understanding of a wide range of strongly correlated 2D systems and moiré quantum materials.

The strain-induced PMFs have important implications for our comprehension of disorder and their impact on strongly correlated states. In particular, twisted bilayer and multilayer graphene are known to be susceptible to twist-angle disorder. Spatial variations in twist angle and strain induce fluctuations in the bandwidth of the flat bands, electron interactions and the emergence of symmetry-broken states^46^. Yet, the effects of the accompanying spatially varying PMFs have not been investigated experimentally. Our findings indicate that the typical reported twist-angle disorder of 0.1° (refs. ^8–11^) can generate *B*_S_ ≈ 0.1 T, markedly influencing magnetotransport behaviour. Resolving LLs in transport at low fields is challenging in twisted devices, potentially arising from such highly spatially varying PMFs. Moreover, in the presence of a magnetic field, the PMF breaks the valley symmetry resulting in different DOS in the two valleys. *B*_S_ fluctuations may thus affect the local Stoner instabilities and symmetry-breaking mechanisms that lead to the quantum anomalous Hall effect, Chern insulators and inhomogeneities in the spontaneous orbital magnetization^18–20^. Finally, the recent development of programmable in-plane bending of graphene ribbons^47^ provides an opportunity for microscale engineering and exploitation of PMFs towards the realization of zero-field quantum Hall and topological insulator-like states^5^ and of all-graphene electronics^48^. The derived method of high-precision determination of local BS and PMF imaging provides a powerful tool for the characterization and optimization of tunable electronic bands and calls for further investigation of the role of strain-induced gauge fields in the formation of symmetry-broken, strongly correlated states of matter.

## Methods

### Device fabrication

The hBN-encapsulated ABA graphene heterostructure was fabricated using the dry-transfer method. The graphene flakes were first exfoliated onto a Si/SiO_2_ (285 nm) substrate. The number of layers in the graphene flakes was determined using Raman microscopy^49^. Then, the hBN (about 30 nm thick) and the graphene flakes were picked up using a polycarbonate on a polydimethylsiloxane dome stamp. The stacks were then released onto a pre-annealed Ti (2 nm)/Pt (10 nm) bottom gate, patterned on the Si/SiO_2_ wafer. The finalized stacks were annealed in a vacuum at 500 °C for strain release^50^. A Ti (2 nm)/Pt (10 nm) top gate was then deposited on top of the stack. The one-dimensional contacts were formed by SF_6_ and O_2_ plasma etching followed by evaporating Cr (4 nm)/Au (70 nm). Then, the device was etched into a Hall bar geometry. Finally, the device was re-annealed at 350 °C in a vacuum. The capacitances per unit area of the bottom and top gates are *C*_bg_ = 0.649 × 10^12^ *e* cm^−2^ V^−1^, *C*_tg_ = 0.668 × 10^12^ *e* cm^−2^ V^−1^. The top and bottom gates are used to control the carrier density *n* = (*C*_bg_*V*_bg_ + *C*_tg_*V*_tg_)/*e* and the effective transverse displacement field *D* = (*C*_tg_*V*_tg_ − *C*_*b*__g_*V*_bg_)/2*ε*_0_, where *ε*_0_ is vacuum permittivity. From fitting the experimental QOs to simulations, we find that *D* = 1 V nm ^−1^ corresponds to the energy difference between the adjacent graphene layers of *Δ*_1_ = 92 meV.

### Transport measurements

Transport characterization of ABA graphene devices was carried out using standard lock-in techniques. The *R*_*xx*_ shows a peak along the diagonal charge neutrality line that increases with *D*, suggesting a gap opening (Extended Data Fig. 1a). The Landau fan shows LL crossings (Extended Data Fig. 1b), consistent with the previous reports^3,37,38,40,51–56^. The QOs from MLG band LLs are visible at low fields, but the BLG LLs can be only resolved above 0.75 T on the electron side and at notably higher fields on the hole doping side (Extended Data Fig. 1c).

### SOT measurements and magnetization reconstruction

The local magnetic measurements were conducted in a custom-built scanning SOT microscope in a cryogen-free dilution refrigerator (Leiden CF1200) at a temperature of 160–350 mK (ref. ^57^). Indium SOT with an effective diameter of about 150 nm and magnetic sensitivity of 20 nT Hz^−1/2^ was fabricated as described previously^33,58,59^. The SOT readout circuit is based on SQUID series array amplifier^60,61^. The SOT is attached to a quartz tuning fork vibrating at about 32.8 kHz (Model TB38, HMI Frequency Technology), which is used as a force sensor for tip height control^62^. The scanning height was about 150 nm above the ABA graphene. An a.c. voltage ${V}_{{\rm{b}}{\rm{g}}}^{{\rm{a}}{\rm{c}}}$ at a frequency of about 1.8 kHz was applied to the bottom gate to modulate the carrier density by ${n}^{{\rm{a}}{\rm{c}}}={C}_{{\rm{b}}{\rm{g}}}{V}_{{\rm{b}}{\rm{g}}}^{{\rm{a}}{\rm{c}}}/e$. A lock-in amplifier was used to measure the corresponding local ${B}_{z}^{{\rm{a}}{\rm{c}}}$ by the scanning SOT. The ${B}_{z}^{{\rm{a}}{\rm{c}}}$ data were symmetrized with respect to the displacement field *D* where applicable. In contrast to other scanning techniques, the magnetic signal is transparent to the metallic top gate, enabling the investigation of a wide range of heterostructures and encapsulated devices.

The 2D ${B}_{z}^{{\rm{a}}{\rm{c}}}(x,y)$ images were used to reconstruct the magnetization *m*_*z*_(*x*, *y*) using the numerical inversion procedure described in ref. ^63^ (Extended Data Fig. 2). As the reconstruction of *m*_*z*_ requires 2D ${B}_{z}^{{\rm{a}}{\rm{c}}}(x,y)$ information, the QOs at a single location or along the one-dimensional line scans are presented in the main text as the raw data of ${B}_{z}^{{\rm{a}}{\rm{c}}}$.

### Magnetic field and modulation amplitude dependence of QOs

The measured signal ${B}_{z}^{{\rm{a}}{\rm{c}}}={n}^{{\rm{a}}{\rm{c}}}({\rm{d}}{B}_{z}/{\rm{d}}n)$ is proportional to the modulation amplitude of the carrier density *n*^ac^ induced by ${V}_{{\rm{b}}{\rm{g}}}^{{\rm{a}}{\rm{c}}}$. It is therefore desirable to use large *n*^ac^ to improve the signal-to-noise ratio. To resolve QOs, however, *n*^ac^ has to be substantially smaller than the period of the oscillations Δ*n*. Extended Data Fig. 2c–e shows the QOs acquired at *B*_a_ = 320 mT using ${V}_{{\rm{b}}{\rm{g}}}^{{\rm{a}}{\rm{c}}}=8\,{\rm{m}}{\rm{V}}$, 35 mV and 100 mV rms corresponding to *n*^ac^ of 5.19 × 10^9^ cm^−2^, 2.27 × 10^10^ cm^−2^ and 6.49 × 10^11^ cm^−2^ rms, respectively. The four-fold degenerate BLG LLs have a period of Δ*n* = 4*B*_a_/*ϕ*_0_ = 3.1 × 10^10^ cm^−2^. The lowest ${V}_{{\rm{b}}{\rm{g}}}^{{\rm{a}}{\rm{c}}}=8\,{\rm{m}}{\rm{V}}$ rms was chosen to result in a peak-to-peak value of *n*^ac^ of 1.47 × 10^10^ cm^−2^, approximately equal to Δ*n*/2 = 1.55 × 10^10^ cm^−2^, which results in an optimal signal-to-noise ratio for detecting the BLG LLs, albeit suppresses the measured ${B}_{z}^{{\rm{a}}{\rm{c}}}/{n}^{{\rm{a}}{\rm{c}}}$ ratio by a factor of π/2. A larger *n*^ac^ washes out the QOs from the BLG LLs, leaving the MLG LLs resolvable as demonstrated in Extended Data Fig. 2d,e. The largest *n*^ac^ also enables observation of the paramagnetic response ∂*M*/∂*μ* = *C*/*ϕ*_0_ in the gap between the zeroth and the first MLG LLs dictated by the Chern number *C* = 2 on the electron side and *C* = −2 on the hole side (Extended Data Fig. 2e).

**Extended Data Fig. 4:**
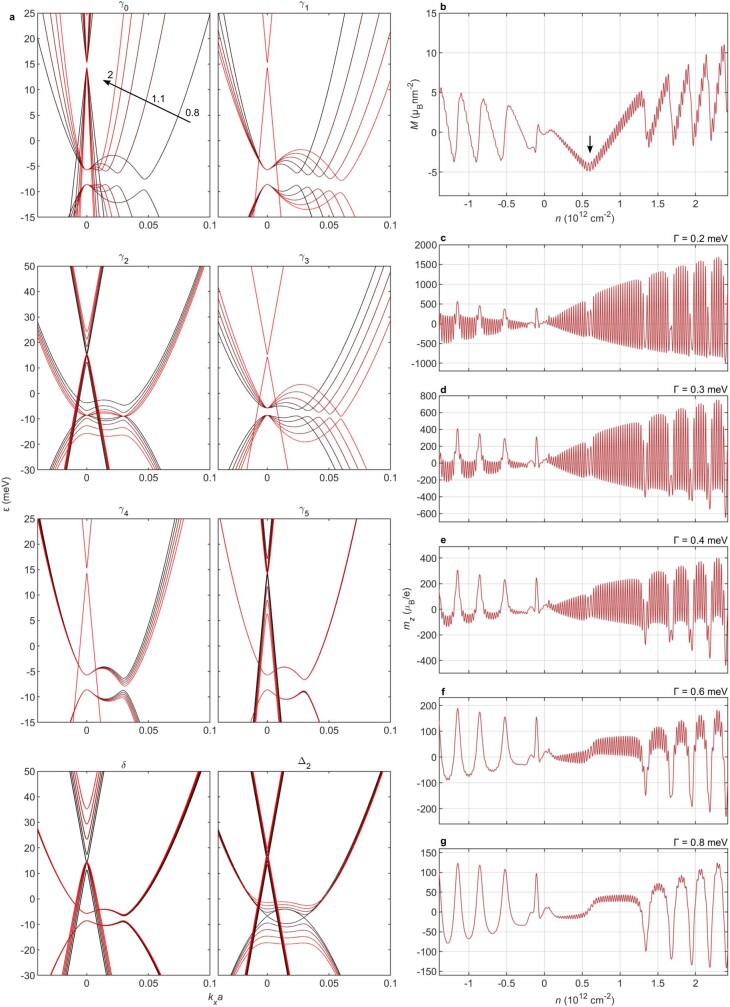
The dependence of the band structure on the SWMc parameters *γ*_0_ to *γ*_5_, *δ*, and *Δ*_2_ at *Δ*_1_ = 0 and suppression of QOs by LL broadening. **a**, The effect of individual parameters on the BS, calculated using the parameters of ref. ^40^ (see Extended Data Table 1) and multiplied by a factor 0.8, 1.1, 1.4, 1.7 and 2 denoted by the different colors from black to red. **b**, Calculated $M\left(n\right)$ at $D$ = 0 V nm^−1^, ${B}_{a}$ = 320 mT, and Dingle parameter $\varGamma$ = 0.3 meV. The V-shaped dip in $M$ (black arrow) corresponds to the McClure peak at the Dirac point of the MLG band^14^. **c**–**g**, Calculated differential magnetization ${m}_{z}=\partial M/\partial n$ for different $\varGamma$ = 0.2 to 0.8 meV. The QOs due to BLG LLs with small energy gaps are suppressed much stronger by $\varGamma$ than the MLG LLs with large gaps.

Extended Data Fig. 2f–h shows the QOs at *B*_a_ = 40 mT, 80 mT and 170 mT. At these low fields, the Dingle broadening greatly suppresses the QOs due to BLG LLs (Extended Data Fig. 4) and reduces the visibility of the MLG LLs at large displacement fields because of the reduction in the gap energies. At 170 mT and ${V}_{{\rm{b}}{\rm{g}}}^{{\rm{a}}{\rm{c}}}=8\,{\rm{m}}{\rm{V}}$ rms, the M2 LLs and the 12-fold degenerate LLs in the gullies are resolved as seen in Extended Data Fig. 2h.

### BS calculations

The BS of ABA graphene was calculated in the tight-binding model following refs. ^2,36^ based on SWMc parameterization^34^. On the basis of {*A*_1_, *B*_1_, *A*_2_, *B*_2_, *A*_3_, *B*_3_}, where *A*_*i*_ and *B*_*i*_ are the two sublattice sites in the *i*th layer, the low-energy effective Hamiltonian can be written as

$${H}_{0}=\left(\begin{array}{cccccc}{\varDelta }_{1}+{\varDelta }_{2} & {v}_{0}{\pi }^{\dagger } & {v}_{4}{\pi }^{\dagger } & {v}_{3}\pi  & {\gamma }_{2}/2 & 0\\ {v}_{0}\pi  & \delta +{\varDelta }_{1}+{\varDelta }_{2} & {\gamma }_{1} & {v}_{4}{\pi }^{\dagger } & 0 & {\gamma }_{5}/2\\ {v}_{4}\pi  & {\gamma }_{1} & \delta -2{\varDelta }_{2} & {v}_{0}{\pi }^{\dagger } & {v}_{4}\pi  & {\gamma }_{1}\\ {v}_{3}{\pi }^{\dagger } & {v}_{4}\pi  & {v}_{0}\pi  & -2{\varDelta }_{2} & {v}_{3}{\pi }^{\dagger } & {v}_{4}\pi \\ {\gamma }_{2}/2 & 0 & {v}_{4}{\pi }^{\dagger } & {v}_{3}\pi  & -{\varDelta }_{1}+{\varDelta }_{2} & {v}_{0}{\pi }^{\dagger }\\ 0 & {\gamma }_{5}/2 & {\gamma }_{1} & {v}_{4}{\pi }^{\dagger } & {v}_{0}\pi  & \delta -{\varDelta }_{1}+{\varDelta }_{2}\end{array}\right)$$

where *Δ*_1_ = −*e*(*U*_1_ − *U*_3_)/2 and *Δ*_2_ = −*e*(*U*_1_ − 2*U*_2_ + *U*_3_)/6, with *U*_*i*_ the potential of layer *i*. *Δ*_1_ is determined by the displacement field, whereas *Δ*_2_ describes the asymmetry of the electric field between the layers. The band velocities *v*_*i*_ (*i* = 0, 3, 4) are related to the tight-binding parameters *γ*_*i*_ by ${v}_{i}\hbar =\frac{\sqrt{3}}{2}{a}_{{\rm{c}}}{\gamma }_{i}$, where *a*_c_ = 0.246 nm is the crystal constant of graphene, *π* = *ξk*_*x*_ + *ik*_*y*_, and *ξ* is the valley index (*ξ* = ±1 for valley K^+^ and K^−^, respectively).

On a rotated basis (*A*_1_ − *A*_3_)/$\sqrt{2}$, (*B*_1_ − *B*_3_)/$\sqrt{2}$, (*A*_1_ + *A*_3_)/$\sqrt{2}$, *B*_2_, *A*_2_, (*B*_1_ + *B*_3_)/$\sqrt{2}$, the Hamiltonian can be rewritten as

$${H}_{{\rm{TLG}}}=\left(\begin{array}{cccccc}{\varDelta }_{2}-\frac{{\gamma }_{2}}{2} & {v}_{0}{\pi }^{\dagger } & {\varDelta }_{1} & 0 & 0 & 0\\ {v}_{0}\pi  & {\varDelta }_{2}+\delta -\frac{{\gamma }_{5}}{2} & 0 & 0 & 0 & {\varDelta }_{1}\\ {\varDelta }_{1} & 0 & {\varDelta }_{2}+\frac{{\gamma }_{2}}{2} & \sqrt{2}{v}_{3}\pi  & -\sqrt{2}{v}_{4}{\pi }^{\dagger } & {v}_{0}{\pi }^{\dagger }\\ 0 & 0 & \sqrt{2}{v}_{3}{\pi }^{\dagger } & -2{\varDelta }_{2} & {v}_{0}\pi  & -\sqrt{2}{v}_{4}\pi \\ 0 & 0 & -\sqrt{2}{v}_{4}\pi  & {v}_{0}{\pi }^{\dagger } & \delta -2{\varDelta }_{2} & \sqrt{2}{\gamma }_{1}\\ 0 & {\varDelta }_{1} & {v}_{0}\pi  & -\sqrt{2}{v}_{4}{\pi }^{\dagger } & \sqrt{2}{\gamma }_{1} & {\varDelta }_{2}+\delta +\frac{{\gamma }_{5}}{2}\end{array}\right)$$

For *Δ*_1_ = 0, the Hamiltonian can be block-diagonalized into MLG-like and BLG-like blocks, that is, *H*_TLG_ = *H*_MLG_ ⊕ *H*_BLG_. A finite displacement field hybridizes the two blocks.

In an external magnetic field, in the Landau gauge, the canonical momentum *π* can be replaced by *π* − *eA*, where *A* is the vector potential. *π* obeys the commutation relation [*π*_*x*_*, π*_*y*_] = −*i*/*l*_*B*_, where ${l}_{B}=\sqrt{\left({\hbar }/{eB}\right)}$ is the magnetic length. As in the usual one-dimensional harmonic oscillator, on the basis of LL orbital |*n*⟩, the matrix elements of *π, π*^†^ are given by

$$\begin{array}{c}{{\rm{K}}}^{+}:\pi | n\rangle =\frac{i\hbar }{{l}_{B}}\sqrt{2(n+1)}| n+1\rangle \\ {\pi }^{\dagger }| n\rangle =-\frac{i\hbar }{{l}_{B}}\sqrt{2n}| n-1\rangle \\ {{\rm{K}}}^{-}:\pi | n\rangle =\frac{i\hbar }{{l}_{B}}\sqrt{2n}| n-1\rangle \\ {\pi }^{\dagger }| n\rangle =-\frac{i\hbar }{{l}_{B}}\sqrt{2(n+1)}| n+1\rangle \end{array}$$

Therefore, the new Hamiltonian can be written on the basis of LL orbitals. Using matrix elements of *π* and *π*^†^ operators, the momentum operators are replaced by raising and lowering the diagonal matrix of dimensions *Λ* × *Λ*, where *Λ* is the cutoff number for the infinite matrix, restricting the Hilbert space with indices *n* ≤ *Λ*. All the other nonzero elements *γ*_*i*_ are substituted by *γ*_*i*_*I*_*Λ*_, where *I*_*Λ*_ is the identity matrix with dimensions *Λ* × *Λ*. As our measurements were performed in low magnetic fields and high-index LLs are often involved, a large cutoff was used so that it spans the energy range significantly larger than in the experiment. We also removed false LLs caused by imposing the cutoff, which usually have very large indices. In the simulations, *Λ* was set to 400 for small carrier-density ranges (Fig. 2) and to 800 for calculations over larger ranges (Figs. 3 and 4).

### Evolution of the BS and LLs with displacement field

Extended Data Fig. 3 shows the calculated BS of ABA graphene using the derived SWMc parameters and the evolution of the LLs with *D* and *B*_a_. At *D* = 0 (*Δ*_1_ = 0), there is essentially no hybridization between the MLG and BLG bands. All the LLs are valley (and spin) degenerate except for the zeroth LLs of the MLG and BLG bands that are valley polarized because of the Berry curvature (Extended Data Fig. 3a). With increasing *Δ*_1_, the gaps of the MLG and BLG bands increase and the hybridization between the bands grows resulting in the formation of mini-Dirac cones (gullies) and in LL anticrossings (Extended Data Fig. 3b–d). At our highest accessible *Δ*_1_ ≈ 50 meV, the lowest LLs in the gullies are well isolated from the rest of the LLs as shown in Extended Data Fig. 3e,f. As the BLG bandgap ${\varDelta }_{{\rm{G}}}^{0}$ is characterized by *C* = 0, it has no magnetization. The six-fold degenerated compressible zeroth LLs ${0}_{{\rm{G}}}^{+}$ and ${0}_{{\rm{G}}}^{-}$ in the gullies also have no magnetization at low fields, *M* = −∂*ε*/∂*B* = 0, because of their zero kinetic energy. As a result, zero magnetization is observed around the CNP over a width of δ*n* = 12*B*_a_/*ϕ*_0_ in carrier density as indicated in Fig. 2c,f. The first paramagnetic signal appears when the Fermi level reaches the *C* = ±6 gaps ${\varDelta }_{{\rm{G}}}^{1}$ and ${\varDelta }_{{\rm{G}}}^{-1}$ between the zeroth and the first gully LLs as shown in Fig. 2f. At elevated magnetic fields, the six-fold gully degeneracy of the zeroth LLs is partially lifted^4,53^.

### Reconstruction of BS parameters

Several experimental studies^3,4,37,38,40,51,52,54,64^ have investigated the tight-binding parameters of ABA graphene as shown in Extended Data Table 1. The high resolution of our data and the fine features attained at low magnetic fields allow high-precision reconstruction of SWMc parameters as follows. We set *γ*_0_ to the standard literature value of 3,100 meV, which corresponds to Fermi velocity of graphene ${v}_{{\rm{F}}}=\frac{\sqrt{3}}{2\hbar }{a}_{{\rm{c}}}{\gamma }_{0}=1{0}^{6}\,{\rm{m}}\,{{\rm{s}}}^{-1}$. The *γ*_0_ sets the overall energy scale, whereas the value of the remaining seven parameters, relative to *γ*_0_, determine the BS. The fitting of the parameters was performed manually. We first determined the effect of the individual parameters on particular features of the BS as shown in Extended Data Fig. 4, which then guided us in the iterative fitting process. In particular, in the absence of displacement field, *Δ*_1_ = 0, the MLG band is affected by only *γ*_0_, *γ*_2_, *γ*_5_ and *δ*, with the gap at the Dirac point given by ${E}_{{\rm{g}}}^{0}=\delta +\frac{{\gamma }_{2}-{\gamma }_{5}}{2}$. The BLG band is strongly dependent on *γ*_0_, *γ*_1_ and *γ*_3_, weakly dependent on *γ*_4_ and essentially independent of *γ*_5_ and *δ*. The BLG gap size is mainly governed by *γ*_2_ and *Δ*_2_. The relative energy shift between the MLG and BLG bands is mainly governed by *γ*_2_.

The dependence of the measured QOs on *n* and *D* at low *B*_a_ provides a very sensitive tool for determining the SWMc parameters. After developing an understanding of the influence of the individual parameters on the relative position of the LLs in specific regions in the (*n*, *D*) plane, an initial set of parameters was chosen to attain an approximate fit to the data. Then fine-tuning of the parameters is achieved by calculating the QOs for each set of parameters and comparing with the data at *D* = 0 V nm^−1^. This process is repeated manually adjusting the different parameters in an iterative manner. After attaining a good fit at *D* = 0, additional fine-tuning was performed to fit the entire range of *D*. As the different parameters have a distinctive effect on the relative positions of the LLs, this manual procedure is readily manageable. The error bars were determined by the values of the individual parameters for which a visible deviation from the data was observed.

The following attributes were particularly informative for the fitting processes:

1. The number of BLG LLs between the adjacent MLG LLs
2. The relative energy shift between MLG and BLG bands
3. LL anticrossings in the gullies
4. The gap size of MLG band

Attribute 1 is determined by the DOS ratio of the two bands, which is predominantly governed by *γ*_1_. By adjusting *γ*_1_ to fit the relative number of BLG and MLG LLs along with optimization of other parameters we obtain *γ*_1_ = 370 ± 10 meV.

Attribute 2 is then used to determine *γ*_2_. The energies of the band extrema and hence the relative position of the zeroth LLs can be calculated analytically. In particular, for *Δ*_1_ = 0, the ${0}_{{\rm{M1}}}^{-}$ LL at the bottom of M1 band is positioned at energy *Δ*_2_ − *γ*_2_/2, whereas the top of BLG valence band is at *Δ*_2_ + *γ*_2_/2. Thus, the relative position between MLG and BLG bands is determined by *γ*_2_ and *Δ*_2_. As the LL spectrum is quite sensitive to *Δ*_2_, *γ*_2_ is determined first. We use the relative position between −1_M3_ and the nearby BLG LLs to fit *γ*_2_, and we get *γ*_2_ = −19 ± 0.5 meV.

Attribute 3 is governed by *γ*_3_, which induces trigonal warping of the BLG bands. As shown in Extended Data Fig. 6, this results in the anticrossings between the BLG LLs and MLG ${0}_{{\rm{M3}}}^{-}$ and ${-1}_{{\rm{M3}}}^{+}$ LLs. From fitting to the experimental data, we obtain *γ*_3_ = 315 ± 10 meV.

Attributes 2 and 4 are used to derive *δ* and *γ*_5_. The MLG band gap at *D* = 0 V nm^−1^ is ${E}_{{\rm{g}}}^{0}=\delta +\left({\gamma }_{2}-{\gamma }_{5}\right)/2$, whereas the gap centre is located at 2*Δ*_2_ + *δ* − (*γ*_2_ + *γ*_5_)/2. In our experimental data, one BLG LL fits within the MLG gap and 20 BLG LLs reside between ${0}_{{\rm{M1}}}^{-}$ and ${-1}_{{\rm{M3}}}$, from which we attain *δ* = 18.5 ± 0.5 meV and *γ*_5_ = 20 ± 0.5 meV. Note that ${E}_{{\rm{g}}}^{0}$ can be either positive or negative. We find that ${E}_{{\rm{g}}}^{0}$ is negative, which means that the zeroth K^−^ LL (${0}_{{\rm{M1}}}^{-}$) resides at the bottom of the M1 band and the zeroth K^+^ LL (${0}_{{\rm{M2}}}^{+}$) is at the top of M2. In this case, the Dirac gap *E*_g_ increases with *Δ*_1_ and the ${0}_{{\rm{M1}}}^{-}$ and the ${0}_{{\rm{M2}}}^{+}$ LLs spread apart with the displacement field as shown in Extended Data Fig. 3f, consistent with experimental data in Fig. 2c,e and calculations in Fig. 2d. If ${E}_{{\rm{g}}}^{0}$ is positive, the zeroth K^−^ LL will reside at the top of M2, whereas the zeroth K^+^ LL will be at the bottom of M1. In this case, on increasing *D*, the Dirac gap closes and then reopens with the crossing of the two zeroth LLs, such that *E*_g_ is always negative at high *D* with zeroth K^−^ LL at the bottom of M1. Extended Data Table 1 shows that the value and the sign of ${E}_{{\rm{g}}}^{0}$ varies notably in the literature. However, only in ref. ^40^ and in the present work the Dirac gap is reported directly. For the rest of the references, the ${E}_{{\rm{g}}}^{0}$ values presented in the table are calculated from the reported values of *δ*, *γ*_2_ and *γ*_5_.

*Δ*_2_ mainly affects the gap of the BLG bands and as −1_M3_ resides closely to the BLG band gap, we use the number of BLG LLs between −1_M3_ and −2_M3_ to fit *Δ*_2_ and get *Δ*_2_ = 3.8 ± 0.05 meV. *γ*_4_ plays the most negligible role, slightly adjusting the shape of the BLG bands. The fitting procedure is to choose these parameters such that the inaccuracy of the number of BLG LLs between any pair of MLG LLs is no more than one. By optimizing all parameters for best fit to the experimental data, we derive *γ*_4_ = 140 ± 15 meV, as shown in Extended Data Table 1.

### Orbital magnetization calculations

Oscillations in orbital magnetization *M* from the LLs can be calculated analytically for either parabolic or Dirac bands as shown previously^65^. However, there is no analytical expression for the LL spectrum in ABA graphene; therefore, the magnetization oscillations have to be calculated numerically. We follow the method described in ref. ^14^ to derive the magnetization *M*(*n*) and then calculate its derivative ∂*M*/∂*n*.

We first consider the case with zero LL broadening. For an arbitrary LL spectrum *E*_*i*_ with degeneracy *D*_*i*_ (*i* is the Landau-level index), the DOS *N*_0_(*ε*) of the system is

$${N}_{0}\left(\varepsilon \right)=\sum _{i}{D}_{i}\delta \left(\varepsilon -{E}_{i}\right).$$

*E*_*i*_ describes spin-degenerate LLs from both valleys with degeneracy ${D}_{i}=2\frac{eB}{h}$. The grand thermodynamic potential *Ω*_0_(*μ*, *B*) is then given by

$${\varOmega }_{0}=-kT{\int }_{-\infty }^{\infty }{N}_{0}\left(\varepsilon \right){\rm{ln}}\left(1+{{\rm{e}}}^{\left[\left(\mu -\varepsilon \right)/kT\right]}\right){\rm{d}}\varepsilon ,$$

where *k* is the Boltzmann constant, *T* is the temperature and *μ* is the chemical potential.

Now we consider LL broadening of width *Γ* (Dingle parameter) with a Lorentzian form

$$L\left(\varepsilon \right)=\frac{1}{\pi }\left(\frac{\varGamma }{{\varepsilon }^{2}+{\varGamma }^{2}}\right).$$

The DOS and the grand potential are then described by

$$N\left(\varepsilon \right)=\sum _{i}{D}_{i}L\left(\varepsilon -{E}_{i}\right),$$

$$\Omega =-kT{\int }_{-\infty }^{\infty }N(\varepsilon ){\rm{ln}}(1+{{\rm{e}}}^{[(\mu -\varepsilon )/kT]}){\rm{d}}\varepsilon =-kT{\int }_{-\infty }^{\infty }\sum _{i}{D}_{i}L(\varepsilon -{E}_{i}){\rm{ln}}(1+{{\rm{e}}}^{[(\mu -\varepsilon )/kT]}){\rm{d}}\varepsilon .$$

Then $M$ is given by

$$M=-\frac{\partial \Omega }{\partial B}=kT{\int }_{-\infty }^{\infty }\sum _{i}(\frac{\partial {D}_{i}}{\partial B}L(\varepsilon -{E}_{i})-{D}_{i}{L}^{{\prime} }(\varepsilon -{E}_{i})\frac{\partial {E}_{i}}{\partial B}){\rm{ln}}(1+{{\rm{e}}}^{[(\mu -\varepsilon )/kT]}){\rm{d}}\varepsilon ,$$

where ${L}^{{\prime} }\left(\varepsilon \right)=\partial L\left(\varepsilon \right)/\partial \varepsilon$. In the zero-temperature limit (*T* → 0), *M* can be simplified:

$$M={\int }_{-\infty }^{\mu }\sum _{i}(\frac{\partial {D}_{i}}{\partial B}L(\varepsilon -{E}_{i})-{D}_{i}{L}^{{\prime} }(\varepsilon -{E}_{i})\frac{\partial {E}_{i}}{\partial B})(\mu -\varepsilon ){\rm{d}}\varepsilon .$$

To compare with our experiment, we need to calculate

$$\frac{\partial M}{\partial n}\left(n\right)=\frac{\partial M}{\partial \mu }\left(n\right)\frac{\partial \mu }{\partial n}\left(n\right),$$

where $\frac{\partial \mu }{\partial n}\left(n\right)$ is the inverse of the DOS as a function of the carrier density and $n(\mu )={\int }_{-\infty }^{\mu }N(\varepsilon ){\rm{d}}\varepsilon$.

**Extended Data Fig. 5:**
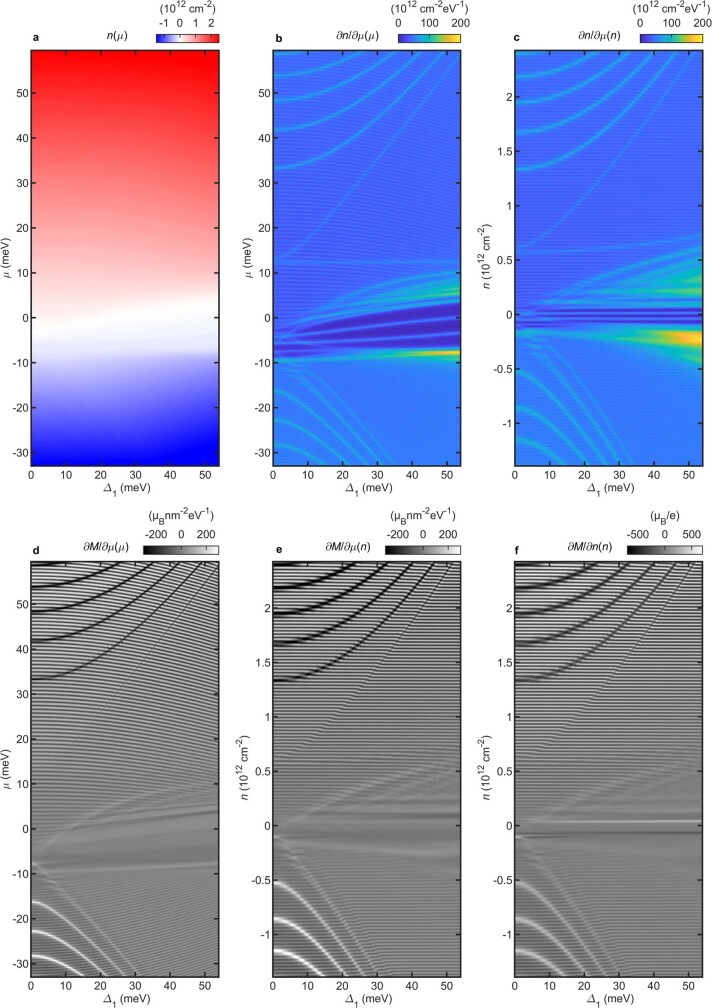
Calculations of the orbital magnetization. **a**, Calculated carrier density $n$ as a function of chemical potential $\mu$ and displacement-field-induced potential difference ${\triangle }_{1}$. **b**, $\partial n/\partial \mu$ versus $\mu$ and ${\triangle }_{1}$. **c**, $\partial n/\partial \mu$ versus $n$ and ${\triangle }_{1}$. **d**, Calculated differential magnetization $\partial M/\partial \mu$ versus $\mu$. **e**,**f**, Calculated $\partial M/\partial \mu$ (**e**) and differential magnetization ${m}_{z}=\partial M/\partial n$ (**f**) versus $n$. *B*_*a*_ = 320 mT and $\varGamma =$ 0.3 meV in all the calculations.

Extended Data Fig. 5 shows the calculated *n*(*μ*), $\frac{\partial n}{\partial \mu }\left(\mu \right)$, $\frac{\partial n}{\partial \mu }\left(n\right)$, $\frac{\partial M}{\partial \mu }\left(\mu \right)$, $\frac{\partial M}{\partial \mu }\left(n\right)$ and $\frac{\partial M}{\partial n}\left(n\right)$ versus *Δ*_1_ at *B*_a_ = 320 mT using the derived SWMc parameters and Dingle broadening *Γ* = 0.3 meV. The modulation in DOS, ∂*n*/∂*μ*, is well resolved in Extended Data Fig. 5b,c, but it is relatively small because of the LL broadening, except near CNP, in which large gaps with vanishing DOS open between the lowest LLs in the gullies at elevated *Δ*_1_.

The calculated ∂*M*/∂*μ* versus *μ* in Extended Data Fig. 5d shows that the crossing of the MLG and BLG LLs does not cause any phase shift. By contrast, in ∂*M*/∂*μ* versus *n* in Extended Data Fig. 5e, the BLG LLs show a 2π shift on crossing the four-fold degenerate MLG LLs and a π shift on crossing the two-fold degenerate zeroth LLs. This arises from the fact that the filling an MLG LL delays filling the next BLG LL versus total *n*, but not versus *μ*. As the DOS modulation $\frac{\partial n}{\partial \mu }\left(n\right)$ is quite small, $\frac{\partial M}{\partial n}\left(n\right)$ in Extended Data Fig. 5f looks very similar to $\frac{\partial M}{\partial \mu }\left(n\right)$ except near CNP.

### Derivation of the Dingle parameter

The energy bands are broadened by the intrinsic broadening *Γ*, given by the quantum scattering time *τ*_q_ = *ħ*/2*Γ*. Hence *Γ* sets the finest meaningful energy resolution with which the band energy can be described. To attain this energy resolution experimentally, we need to use the lowest *B*_a_ for which the LL energy gaps are comparable to *Γ*. In this limit, the amplitude of the QOs is rapidly suppressed with increasing *Γ*. Extended Data Fig. 4c–g shows the calculated QOs for various Dingle parameters *Γ* = 0.2–0.8 meV. As the energy spacing of the BLG LLs in the conduction band is about 1 meV, the amplitude of their QOs is suppressed by about two orders of magnitude over this range of *Γ*, whereas in the valence band, in which the LL gaps are about 0.6 meV, the QOs are completely quenched with the higher *Γ*. By contrast, the amplitude of the QOs of the MLG LLs, which have an order of magnitude larger gaps at low carrier densities, is much less affected by these *Γ* values. As a result, the relative amplitude of the MLG and BLG QOs is strongly dependent on *Γ*, enabling its accurate determination. By fitting to the experimental data in Fig. 2, we obtain *Γ* *=* 0.3 ± 0.05 meV, which also provides a very good agreement in quantitative comparison between the amplitudes of the measured ${B}_{z}^{{\rm{a}}{\rm{c}}}$ and the calculated *m*_*z*_ taking into account the 2D magnetization reconstruction.

The finite *n*^ac^ modulation by ${V}_{{\rm{b}}{\rm{g}}}^{{\rm{a}}{\rm{c}}}$ also causes a suppression of the apparent amplitude of the QOs. It can be shown that if the peak-to-peak value of the carrier-density modulation is less than half of the LL degeneracy, *n*^ac^ < 2*B*_a_/*ϕ*_0_, which is the case in our high-resolution measurements, the suppression is less than a factor of π/2. For larger *n*^ac^,  the visibility is suppressed rapidly as shown in Extended Data Fig. 2d,e. In particular, in Figs. 3 and 4 we have intentionally used larger *n*^ac^  to suppress the QOs due to BLG LLs and to improve the signal-to-noise ratio for detections of the MLG LLs. As this type of suppression of the apparent amplitude of QOs is harder to simulate in our BS calculations, we have used *Γ* = 0.3 meV for the calculations presented in all the figures except in Figs. 3 and 4, where *Γ* = 0.6 meV was used instead for suppression of the BLG QOs artificially. This larger *Γ*  does not affect the shape of the calculated MLG QOs appreciably but reduces their amplitude.

Our derived *Γ* = 0.3 meV with corresponding local quantum scattering time *τ*_*q*_ *=* *ħ*/2*Γ* ≈ 1 ps, is about four times lower than the value reported based on global SdH oscillations^40^. This is consistent with the observation that the lowest magnetic field for detection of QOs in our local dHvA measurements is substantially lower than what is required for detection of the SdH oscillations (Extended Data Fig. 1). The large *Γ* reported based on SdH oscillations is probably because of sample inhomogeneity, such as charge disorder and the PMFs (*B*_S_). Hence, the measurement of the local dHvA QOs enables the determination of the local BS with energy resolution set by the intrinsic broadening *Γ* of the energy bands. This is of key importance for the study of BS of twisted vdW materials that are particularly prone to strain and spatial inhomogeneities.

### LL anticrossings

**Extended Data Fig. 6:**
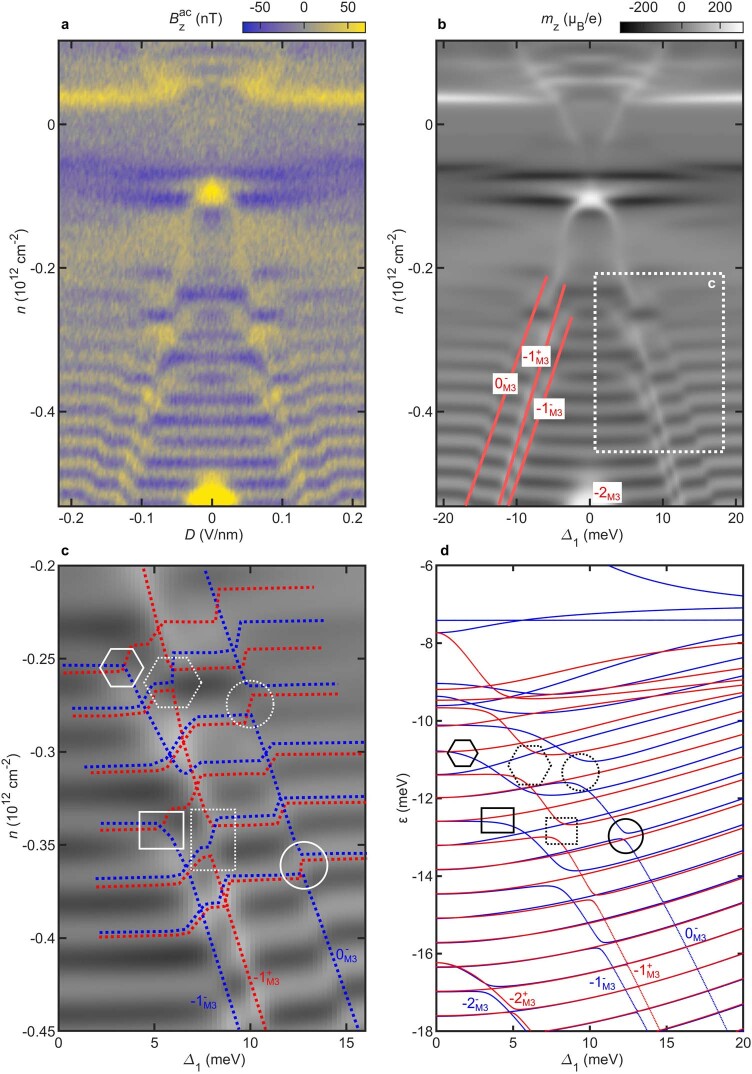
Landau level anticrossings. **a**, Measured QOs in ${B}_{z}^{a{\rm{c}}}$ versus $n$ and $D$ at ${B}_{a}=$ 320 mT near the top of the BLG valence band reproduced from Fig. 2f. **b**, Calculated ${m}_{z}$ using the derived SWMc parameters providing an excellent fit to the experimental data. **c**, A zoom-in of the region marked in **b** with overlaid schematic LLs. **d**, Calculated LLs in the ${{\rm{K}}}^{-}$ (blue) and ${{\rm{K}}}^{+}$ (red) valleys. The open symbols denote the points of valley splitting and LL anticrossings between the MLG and BLG LLs. The splitting of ${-1}_{M3}$ LL into valley polarized ${-1}_{M3}^{-}$ and ${-1}_{M3}^{+}$ LLs is clearly resolved in calculations and in the experimental data.

The hybridization between the BLG and MLG bands on increasing *Δ*_1_ with the displacement field gives rise to partial lifting of valley degeneracy of the LLs. This effect is particularly pronounced near the top of the BLG valence band at intermediate values of *Δ*_1_ as shown in Extended Data Fig. 6c,d. Here, when MLG and BLG LLs in the same valley intersect, the strong band hybridization and non-vanishing *γ*_3_ leads to avoided crossing between the LLs as marked by the open symbols. Interestingly, the anticrossing occurs between the MLG LLs and every third BLG LL. Our derived SWMc parameters provide an excellent fit to the experimentally observed anticrossings as demonstrated in Extended Data Fig. 6a,b. Moreover, the strong hybridization lifts the valley degeneracy of the first MLG LL in the M3 sector as shown by the pronounced splitting between ${-1}_{{\rm{M3}}}^{-}$ and ${-1}_{{\rm{M3}}}^{+}$ in Extended Data Fig. 6b–d. This splitting is resolved experimentally in Extended Data Fig. 6a.

### Interference of BLG LLs

**Extended Data Fig. 7:**
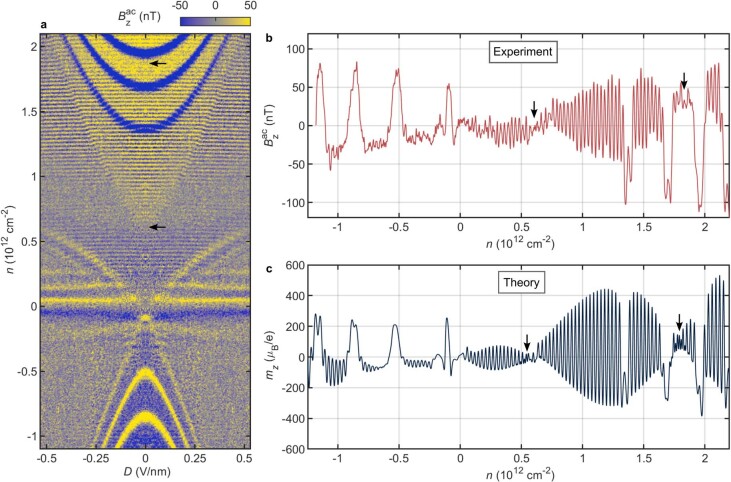
Beating of the BLG LLs. **a**, QOs versus $n$ and $D$ at ${B}_{a}$ = 320 mT acquired at site B in Fig. 3b using ${V}_{{bg}}^{{ac}}$ = 8 mV rms. **b**, ${B}_{z}^{{ac}}$ versus $n$ profile at $D$ = 0 V nm^−1^. **c**, Calculated QOs with ${B}_{S}$ = 4.2 mT. The black arrows indicate the positions of the beating nodes.

The interference of the LLs can be observed also in the BLG bands at the same locations at which it is present in the MLG bands. Extended Data Fig. 7 shows the QOs acquired at site B as in Figs. 3h and 4e, but using lower ${V}_{{\rm{b}}{\rm{g}}}^{{\rm{a}}{\rm{c}}}=8\,{\rm{m}}{\rm{V}}$ rms that enables resolving the BLG LLs. The beating nodes at around 0.5 × 10^12^ cm^−2^and 1.8 × 10^12^ cm^−2^ are seen (Extended Data Fig. 7b), which can be well reproduced by the simulations using *B*_S_ = 4.2 mT (Extended Data Fig. 7c).

### Resolution of the PMF by LL interference

The minimal PMF that can be measured using the interference method is determined by the highest accessible LL index of the beating node ${N}_{{\rm{b}}}^{1}$. At *B*_a_ = 320 mT in the accessible range of *n*, the highest MLG LL index in ABA graphene is ±70, and hence the minimal ${B}_{{\rm{S}}}={B}_{{\rm{a}}}/\left(4{N}_{{\rm{b}}}^{1}\right)=1.14\,{\rm{mT}}$. For comparison, the lowest PMF that has been recently resolved by scanning tunnelling microscope is *B*_S_ ≈ 0.5 T (ref. ^66^).

### PMFs on different length scales

In moiré 2D materials, notable lattice relaxation occurs, giving rise to periodic strain and PMFs up to tens of tesla within moiré unit cell^67–69^. This short-range periodic PMF is part of the periodic potential that determines the BS^70,71^, but does not affect the usual LLs. By contrast, the strain that we probe varies gradually on a much larger length scale (about 1 µm). This strain gives rise to smooth PMFs, which shift the LLs in the presence of *B*_a_ and form strain-induced LLs at zero magnetic field^6,72–75^.

### Towards characterization and use of PMFs

Strain engineering has been proposed to realize programmable PMFs leading to topological phases and various electronic devices^5,48^. Although large, short-range PMFs have been widely observed^6,7,67–69,72–75^, long-range homogeneous and controllable PMFs required for the development of new functionalities and valleytronics have not been realized^5,43,44^. Several methods have been proposed to induce variable mesoscale strain, including bending, MEMS, piezoelectric devices and polyimide deformation^47,76–79^, but the generated PMFs could not be detected. Our method enables the integration of such in situ controllable strain engineering, transport measurements and high-resolution local PMF imaging, laying the groundwork for investigation and use of PMFs.

### Discussion of possible alternative mechanisms of interference of QOs

We consider below several other possible mechanisms that can alter the BS and induce degeneracy lifting, which may lead to interference of the LLs, and show that they are incompatible with the experimental data.

#### Band shifting

**Extended Data Fig. 8:**
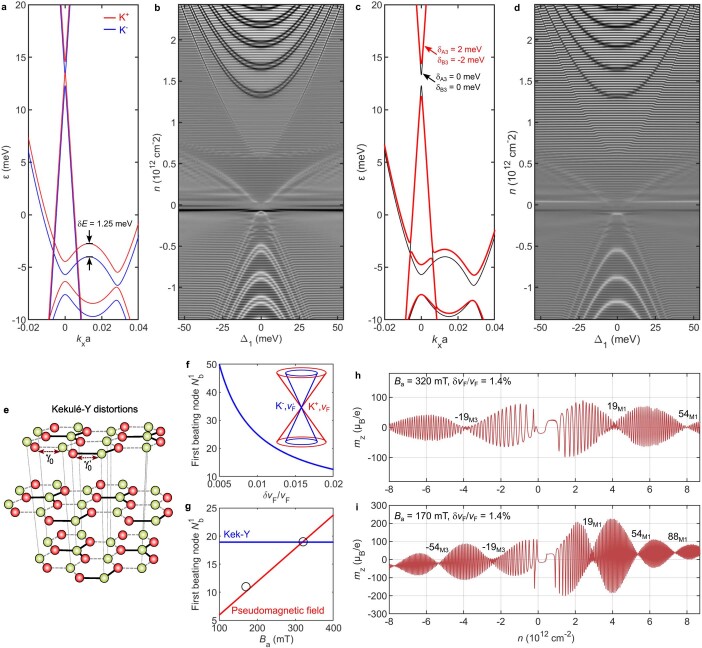
Alternative mechanisms of LL interference. **a**, Band structure of ABA graphene with a relative energy shift of $\delta E$ = 1.25 meV between the ${{\rm{K}}}^{+}$ and ${{\rm{K}}}^{-}$ bands. **b**, The corresponding calculated QOs show splitting of the lowest MLG LLs inconsistent with the experimental data. **c**, Band structure of ABA graphene with (red) and without (black) staggered sublattice potential ${\delta }_{A3}$ = 2 meV, ${\delta }_{B3}=-$2 meV. **d**, Corresponding calculated QOs showing no LL interference. **e**, Kekulé-Y distortion with ${\gamma }_{0}^{{\prime} }$ hopping parameter along the bonds emphasized in black. **f**, The dependence of the first beating node ${N}_{b}^{1}$ on the difference of the Fermi velocities ${\triangle v}_{F}/{v}_{F}$ of the two valleys. Inset, schematic of the MLG band dispersions of the two valleys. **g**, The dependence of ${N}_{b}^{1}$ on ${B}_{a}$ for Kekulé-Y distortion (blue), PMF (red), and the experimental data (circles). **h**, The calculated QOs with ${\triangle v}_{F}/{v}_{F}$ = 1.4% at ${B}_{a}$ = 320 mT. **i**, Same as **h** at ${B}_{a}$ = 170 mT.

**Extended Data Fig. 9:**
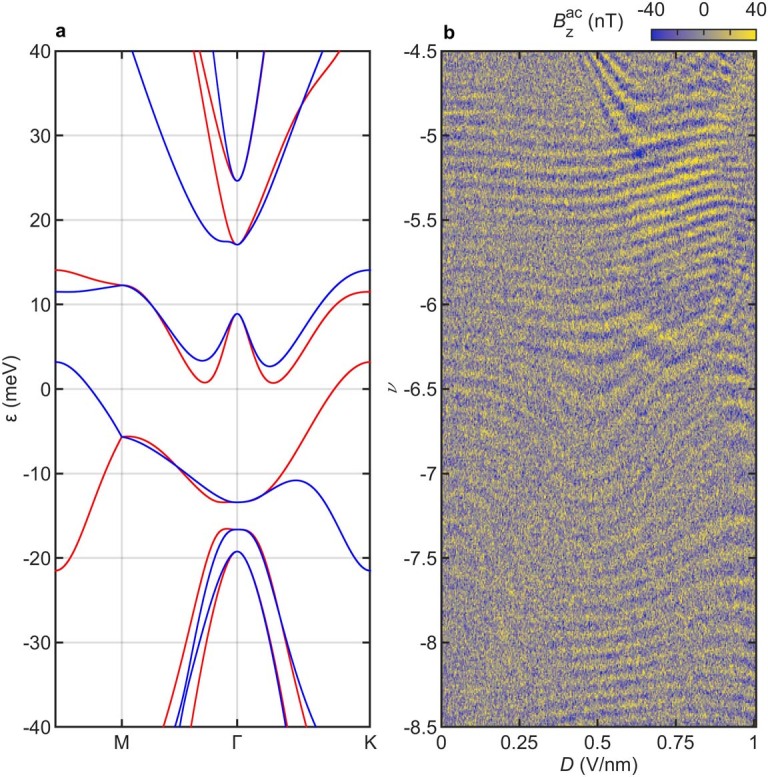
Band structure and QOs in twisted double bilayer graphene (TDBLG). **a**, Calculated single-particle BS of TDBLG under an electrostatic potential difference of 30 meV, which causes an overlap between the valence flat and dispersive bands. **b**, QOs measured in TDBLG as a function of moiré filling factor ${\rm{\nu }}$ and the displacement field *D* at 310 mT. The evolution of the flat and dispersive bands with *D* gives rise to very complicated QO patterns, which exemplifies the applicability of the dHvA technique to a broad range of vdW materials.

Spin–orbit coupling as well as the Zeeman effect at elevated fields can lift flavour degeneracy producing an energy shift between the bands of opposite spin or valley. Both the intrinsic spin–orbit coupling in graphene and the Zeeman contributions at our low magnetic fields result in a negligible energy shift of the order of µeV (refs. ^80,81^), which cannot account for the experimental data. Nevertheless, we explore whether a generic rigid shift between bands can reproduce the revealed LL interference pattern. In Fig. 3h, the first node in the interference of the MLG LLs occurs at an index *N* ≈ 19. The corresponding LL energy gap is $\triangle {E}_{N}={E}_{N+1}-{E}_{N}=\sqrt{2e\hbar {v}_{{\rm{F}}}^{2}{B}_{{\rm{a}}}}\left(\sqrt{N+1}-\sqrt{N}\right)\approx 2.5\,{\rm{meV}}$. For the destructive interference, the LLs of the two bands have to be out of phase, namely, shifted by *δE*_*N*_ ≈ 1.25 meV. Extended Data Fig. 8a shows the BS with a rigid shift of 1.25 meV between the K^+^ and K^−^ bands with the corresponding calculated QOs presented in Extended Data Fig. 8b. The main resulting feature is that the MLG LLs are split into two, which is markedly different from the experimental QOs. This points out that to reproduce the observed QOs, the energy shift *δE*_*N*_ between the interfering LLs has to grow with the LL index rather than being constant or decreasing with *N*. This is the behaviour in the case of PMF, where $\delta {E}_{N}=\sqrt{2e\hbar {v}_{{\rm{F}}}^{2}N}\left(\sqrt{{B}_{{\rm{a}}}+{B}_{{\rm{S}}}}-\sqrt{{B}_{{\rm{a}}}-{B}_{{\rm{S}}}}\right)\approx {B}_{{\rm{S}}}\sqrt{2e\hbar {v}_{{\rm{F}}}^{2}N/{B}_{{\rm{a}}}}$ grows as $\sqrt{N}$.

#### Staggered substrate potential

The possible alignment between the hBN and ABA graphene can cause an on-site potential difference between the A and B sublattices. Here we consider the simplest situation in which one of the graphene layers (bottom) is aligned with the hBN giving rise to a staggered substrate potential. In this case, the Hamiltonian can be written on the basis of {*A*_1_, *B*_1_, *A*_2_, *B*_2_, *A*_3_, *B*_3_} as

$${H}_{0}=\left(\begin{array}{cccccc}{\varDelta }_{1}+{\varDelta }_{2} & {v}_{0}{\pi }^{\dagger } & {v}_{4}{\pi }^{\dagger } & {v}_{3}\pi  & {\gamma }_{2}/2 & 0\\ {v}_{0}\pi  & \delta +{\varDelta }_{1}+{\varDelta }_{2} & {\gamma }_{1} & {v}_{4}{\pi }^{\dagger } & 0 & {\gamma }_{5}/2\\ {v}_{4}\pi  & {\gamma }_{1} & \delta -2{\varDelta }_{2} & {v}_{0}{\pi }^{\dagger } & {v}_{4}\pi  & {\gamma }_{1}\\ {v}_{3}{\pi }^{\dagger } & {v}_{4}\pi  & {v}_{0}\pi  & -2{\varDelta }_{2} & {v}_{3}{\pi }^{\dagger } & {v}_{4}\pi \\ {\gamma }_{2}/2 & 0 & {v}_{4}{\pi }^{\dagger } & {v}_{3}\pi  & -{\varDelta }_{1}+{\varDelta }_{2}+{\delta }_{A3} & {v}_{0}{\pi }^{\dagger }\\ 0 & {\gamma }_{5}/2 & {\gamma }_{1} & {v}_{4}{\pi }^{\dagger } & {v}_{0}\pi  & \delta -{\varDelta }_{1}+{\varDelta }_{2}+{\delta }_{B3}\end{array}\right).$$

For concreteness, we choose *δ*_*A*__3_ = 2 meV and *δ*_*B*__3_ = −2 meV. The resulting BS is shown in Extended Data Fig. 8c (red) in comparison with the original BS (black). The staggered substrate potential increases the gaps of the MLG and BLG bands but does not lift the valley degeneracy and therefore does not lead to beating. Extended Data Fig. 8d presents the calculated QOs showing no LL beating.

#### Kekulé distortion

Kekulé distortions are the bond density waves that have been observed in graphene epitaxially grown on copper^82^ or in the presence of strain^83^. In contrast to the O-type Kekulé distortion that opens a gap at the Dirac point, we find that the Y-type^84^ distortion can result in LL interference. The Y-shaped modulation of the bond strength, parametrized by the hopping parameters *γ*_0_ and $${\gamma }_{0}^{{\prime} }$$ (Extended Data Fig. 8e), gives rise to valley-momentum locking and to inequivalent Fermi velocities for both the MLG and BLG bands. Hence, it lifts the valley degeneracy of the LLs resulting in chiral symmetry breaking. In the SWMc model, *γ*_0_ is the sole parameter that controls the Fermi velocity *v*_F_ of the MLG band ($${v}_{{\rm{F}}}=\frac{\sqrt{3}}{2}\frac{a{\gamma }_{0}}{\hbar }$$). The energy difference between the LLs from the two valleys with the same index *N* is $$\delta {E}_{N}=\sqrt{2e\hbar N{B}_{{\rm{a}}}}{\Delta v}_{{\rm{F}}}$$, where $${\Delta v}_{{\rm{F}}}=\frac{\sqrt{3}}{2}\frac{a}{\hbar }({\gamma }_{0}-{\gamma }_{0}^{{\prime} })$$. The first beating node appears when *δE*_*n*_ is equal to half of the gap size: $$\sqrt{2e\hbar N{B}_{{\rm{a}}}}{\Delta v}_{{\rm{F}}}\,=$$$$\sqrt{e\hbar {v}_{{\rm{F}}}^{2}{B}_{{\rm{a}}}/2N}/2$$, which yields $${N}_{{\rm{b}}}^{1}={v}_{{\rm{F}}}/\left(4{\Delta v}_{{\rm{F}}}\right)$$ as shown in Extended Data Fig. 8f. In Fig. 3h, $${N}_{{\rm{b}}}^{1}=19$$, which corresponds to a very weak Kekulé distortion with Δ*v*_F_/*v*_F_ = 1.4 × 10^−2^. However, the Kekulé distortion results in $${N}_{{\rm{b}}}^{1}$$ that is independent of *B*_a_ as corroborated by the calculated QOs for *B*_a_ =  320 mT and 170 mT in Extended Data Fig. 8h,i. This is because the LLs shift in the same proportion in the two valleys with *B*_a_. This is in sharp contrast to beating due to PMF for which $${N}_{{\rm{b}}}^{1}={B}_{{\rm{a}}}/\left(4{B}_{{\rm{S}}}\right)$$ is proportional to *B*_a_. The experimental data points in Extended Data Fig. 8g (circles) are consistent with PMF and incompatible with the Kekulé distortion.

#### Disorder in BS parameters

The BS can vary in space because of various types of disorder. Focusing on the Dirac bands, for example, the energy of the Dirac point or *v*_F_ could be position dependent without breaking the valley symmetry. If the parameters change gradually in space on lengths scale larger than our spatial resolution of about 150 nm, the LLs will shift gradually in space following the variations in the BS without showing interference at any location. Let us now consider the opposite case of sharp boundaries between domains with different BS. In this situation, at the boundaries, the finite size of our SOT may result in the simultaneous detection of LLs originating from the two neighbouring domains giving rise to apparent interference. In such a case, we expect to observe interference along a network of grain boundaries with width comparable to our SOT size. Instead, Fig. 3e shows well-defined domains of typical width of 1 µm and length of up to 2 µm, much larger than the SOT size, over which the interference is rather uniform. Furthermore, most of the domains showing beating are located at the ends or corners of the device, so they do not have two neighbouring domains that can cause the apparent interference. Finally, if there is a relative shift in the Dirac point between the neighbouring domains, the apparent interference patterns at the boundary would evolve similar to that calculated in Extended Data Fig. 8b, whereas if *v*_F_ changes between the domains the beating node ${N}_{{\rm{b}}}^{1}$ of the apparent interference would be independent of *B*_a_ as calculated in Extended Data Figs. 8f–i. Both these possibilities are inconsistent with the experimental data. More generally, the *B*_a_ dependence of the LL interference due to variations in BS is distinctly different from the one caused by *B*_S_. We therefore conclude that disorder that causes spatial variations in BS without creating PMFs cannot explain the observed LL interference.

## Online content

Any methods, additional references, Nature Portfolio reporting summaries, source data, extended data, supplementary information, acknowledgements, peer review information; details of author contributions and competing interests; and statements of data and code availability are available at 10.1038/s41586-023-06763-5.

## Figures and Tables

**Extended Data Table 1:**
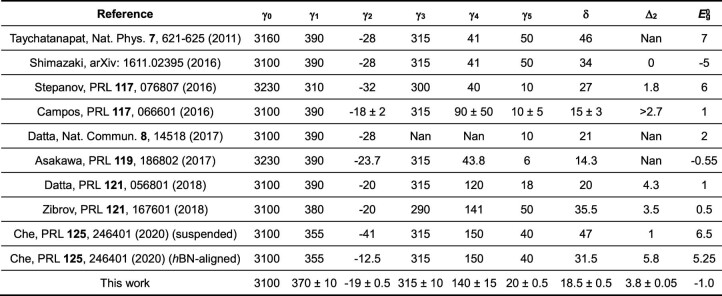
Comparison of SWMc parameters in different works

The parameters are in units of meV.

## Data Availability

The data that support the findings of this study are available from the corresponding authors on reasonable request.

## Code availability

The BS calculations codes used in this study are available from the corresponding authors on reasonable request.
